# An enzyme's metal preference evolves through redox modulation driven by the cofactor's secondary coordination sphere

**DOI:** 10.1093/molbev/msag040

**Published:** 2026-02-13

**Authors:** Eilidh S Mackenzie, Kacper M Sendra, Arnaud Baslé, Rafał Mazgaj, Thomas E Kehl-Fie, Kevin J Waldron

**Affiliations:** Biosciences Institute, Faculty of Medical Sciences, Newcastle University, Newcastle upon Tyne NE2 4HH, UK; Biosciences Institute, Faculty of Medical Sciences, Newcastle University, Newcastle upon Tyne NE2 4HH, UK; Biosciences Institute, Faculty of Medical Sciences, Newcastle University, Newcastle upon Tyne NE2 4HH, UK; Institute of Biochemistry and Biophysics, Polish Academy of Sciences, Pawińskiego 5a, Warsaw 02-106, Poland; Department of Microbiology & Immunology, University of Iowa, Iowa City, IA 52242, USA; Biosciences Institute, Faculty of Medical Sciences, Newcastle University, Newcastle upon Tyne NE2 4HH, UK; Institute of Biochemistry and Biophysics, Polish Academy of Sciences, Pawińskiego 5a, Warsaw 02-106, Poland

**Keywords:** metalloenzyme, protein evolution

## Abstract

Changes in protein properties and functions are central to the evolution of life. Metalloproteins can evolve by changing their preference from one metal cofactor to another. Recently, we demonstrated that the widely distributed iron- or manganese-dependent superoxide dismutase (SodFM) family has undergone numerous metal-preference changes, including during evolutionary adaptation of pathogenic bacteria to altered metal availability within the host. Yet the underlying properties of metal-binding sites that control metalloenzyme metal preference are unclear, and thus, we lack an understanding of how enzymatic metal preference can be reshaped by evolution. Here, we used spectral features of bound iron or manganese, whose intensities reflect their oxidation state, to assess how their redox properties are tuned during SodFM evolution. We systematically analyzed the metal oxidation state across diverse SodFMs from multiple phylogenetic groups with different catalytic metal preferences, including those known to have undergone evolutionary metal-preference switching. We observed a striking relationship between resting oxidation state and catalytic metal preferences. Mutagenesis of second-sphere residues previously identified as determining metal preference revealed that they modulate metal-dependent activity and cofactor oxidation state in tandem, demonstrating these properties are linked. Together, these data argue that the differing SodFM metal preferences observed across the tree of life evolved through tuning of their redox properties by the secondary coordination sphere. This study gives insight into the process by which a metalloenzyme originally optimized for one metal cofactor can evolve a new metal preference, under suitable selection pressure, through re-optimization of its active site for catalytic reactivity of the new metal cofactor.

## Introduction

The ability of proteins to change their properties and functions is fundamental to the evolution of life. For example, evolutionary changes in metalloenzymes can enable them to utilize a new metal cofactor while retaining their ancestral activity or can facilitate acquisition of entirely new catalytic functions (Valasatava et al. 2018; Barwinska-Sendra et al. 2020; Sendra et al. 2023). Almost half of all enzymes utilize metal cofactors, reflecting their ancient and essential recruitment by biology (Kirschvink et al. 2000; Waldron et al. 2009; Dupont et al. 2010). Yet the biophysical mechanisms by which protein metal utilization evolves remain to be elucidated.

We recently established the iron (Fe) or manganese (Mn) superoxide dismutase (SOD) enzyme family (SodFM) as a model system for studying metal-preference evolution (Sendra et al. 2023). SODs utilize redox-active metal ions to catalyze detoxification of the reactive oxygen species superoxide and play critical roles in human health, including the ability of microbes to survive within the host during infection (Sheng et al. 2014; Garcia et al. 2017). SodFMs are the most prevalent SOD type across the tree of life (Sendra et al. 2023). Contrary to previous classification of SodFMs into three distinct subtypes according to their metal specificity (Parker and Blake 1988; Kirschvink et al. 2000; Wintjens et al. 2004; Sheng et al. 2014), we demonstrated their catalysis displays a spectrum of metal preference, ranging from strongly Mn-preferring, through metal-interchangeable (cambialistic), to strongly Fe-preferring (Barwinska-Sendra et al. 2020; Sendra et al. 2023). Importantly, catalytic metal preference is not constrained within SodFM phylogenetic groups (Sendra et al. 2023). Four of the five identified SodFM subfamilies (denoted SodFM1-5) contain isozymes with varying metal preference, demonstrating their metal utilization is evolutionarily dynamic (Sendra et al. 2023). Multiple independent SodFM metal-preference changes have occurred, ranging from very ancient to much more recent evolutionary modulations. Importantly, cambialistic SodFMs that exhibit activity with either cofactor have likely evolved from more metal-specific ancestors on multiple independent occasions. The most recent identified emergence of cambialism occurred during the proposed neofunctionalization of a duplicated SodFM in *Staphylococcus aureus* (Barwinska-Sendra et al. 2020), which enabled this pathogenic bacterium to circumvent the Mn deprivation it experiences during infection imposed by host nutritional immunity (Garcia et al. 2017). Thus, evolution manipulates the metal preference of SodFMs, and these changes can occur on relatively short evolutionary timescales (Barwinska-Sendra et al. 2020; Sendra et al. 2023).

The cofactor flexibility of cambialistic SodFMs challenges the assumption that oxidoreductase metalloenzymes are highly specific for their cognate metal (Foster et al. 2014; Imlay 2014; Valdez et al. 2014). Their presence in diverse organisms, including pathogenic microbes, emphasizes our limited understanding of metalloenzyme metal utilization and the need to address this gap in knowledge. Crucially, despite their different metal preferences, all SodFMs are related in sequence and share a conserved architecture, including the structure of their metal-binding active site (Steinman and Hill 1973; Stallings et al. 1984; Lah et al. 1995; Sugio et al. 2000). Amino acids within the metal's secondary coordination sphere, including a key residue denoted X_D-2_ (reflecting its position in the primary sequence relative to the conserved metal-coordinating aspartate), have frequently been involved in these evolutionary metal-preference modulation events (Barwinska-Sendra et al. 2020; Sendra et al. 2023). However, it is unclear how such minor mutations can drive the biochemical changes observed during SodFM evolution.

Although other models have been proposed (Whittaker and Whittaker 1997; Edward et al. 1998; Yamakura et al. 2003), a “redox tuning” model has gained some limited empirical support as an explanation of the differences in metal preference between the *Escherichia coli* MnSOD and FeSOD (Vance and Miller 1998b). This model hypothesizes that these SodFM isozymes differentially manipulate the intrinsic reduction potentials of Fe^2+^/Fe^3+^ (*E*^0^ = 0.77 V) and Mn^2+^/Mn^3+^ (*E*^0^ = 1.51 V) to be close to the optimal potential for catalyzing both steps of the chemical reaction (*E*^0^ ≈ 0.3 V) with its cognate ion, with MnSOD having to apply a greater adjustment than FeSOD to achieve this optimum (Vance and Miller 1998a; Miller 2008). The resulting mismatch of tuning could explain why each of these SodFM isozymes has high turnover with one metal but is catalytically ineffective with the other metal, as the “wrong” metal's reduction potential will be inappropriate within an architecture optimized for the “correct” metal (Vance and Miller 1998a; Vance and Miller 2001). Specifically, the MnSOD architecture would “over-tune” the potential when loaded with Fe, and the FeSOD architecture would “under-tune” bound Mn. The model also predicts that cambialistic SodFMs should be suboptimally tuned but within the target range for both metals and therefore should exhibit lower turnover than metal-preferring SodFMs. This would suggest that cambialism is an evolutionary trade-off, compromising higher turnover in favor of physiological flexibility from relaxed cofactor preference. When a SodFM evolves to utilize the alternative metal, thereby switching its metal preference, such a model would require evolutionary retuning of the reduction potential.

Direct testing of the redox tuning model is challenging because quantitative measurement of SodFM reduction potentials is not facile with existing methods (Lawrence and Sawyer 1979; Verhagen et al. 1995; Lévêque et al. 2001). To overcome these limitations, here we leveraged the spectral features of their bound *d*-block metals, Fe and Mn, whose intensities reflect their oxidation state, to assess the distribution of redox states of the population of metal ions in SodFM samples. We performed a systematic, semiquantitative analysis of cofactor oxidation state under standardized assay conditions across a vast set of purified samples of diverse SodFM isozymes, from multiple phylogenetic groups and with a range of catalytic metal preferences. Our sampling included SodFMs known to have undergone evolutionary metal-preference switching and a set of mutated variants whose preference had been artificially altered. We observed a striking trend in their redox state that followed their catalytic metal preferences, consistent with tuning of the metal's redox properties by the protein architecture being an underlying biophysical principle of SodFM metal-preference evolution.

## Results

### Spectral properties of SodFMs at aerobic equilibrium reflect their metal preference

A set of seven bacterial SodFMs were initially selected for characterization from the two main subfamilies, SodFM1 and SodFM2. We previously identified evolutionary metal-preference modulation events in both of these subfamilies (Sendra et al. 2023). Each enzyme was purified to homogeneity in both Fe- and Mn-loaded forms; in triplicate, their metal content was confirmed by inductively coupled plasma mass spectrometry (ICP-MS), and the metal preference of its enzymatic activity was quantitatively determined. A cambialism ratio (CR), defined as the Fe-dependent activity divided by the Mn-dependent activity (Barwinska-Sendra et al. 2020) (Table 1), was experimentally verified for each enzyme. This ratio approaches 0 for increasingly Mn-preferring SodFMs, has values close to 1 for cambialistic enzymes capable of utilizing either metal with similar efficiency, and increases >2 with increasingly Fe-preferring activity. All CR values were calculated for metal-verified protein preparations and corroborated the previously measured approximate CR (aCR) values, estimated using a standardized 24-well plate format activity assay in soluble extracts of *E. coli* cells expressing the heterologous SodFM isozymes (Sendra et al. 2023).

**Table 1:** Summary of CR and nOS data of all wild-type SodFMs and mutant variants under study.

Species	Wild type					X_D-2_-X_D-1_ mutant			
	Enzyme	SodFM	CR^a^	nOS_Fe_^b^	nOS_Mn_^b^	Mutation	CR^a^	nOS_Fe_^b^	nOS_Mn_^b^
*E. coli*	*Ec*SodA^c^	FM1	ND^c^	ND^c^	0.64	G_D-2_T-L_D-1_V^c^	ND^c^	ND^c^	0.47
*S. aureus*	*Sa*SodA	FM1	0.09	0.06	0.53	G_D-2_L-L_D-1_F	0.49	0.16	0.27
*B. subtilis*	*Bs*SodA	FM1	0.13	0.09	0.56	G_D-2_V-L_D-1_I	1.49	0.23	0.24
*B. anthracis*	*Ba*SodA1	FM1	0.20	0.01	0.62	G_D-2_V-L_D-1_I	0.74	0.21	0.21
*L. monocytogenes*	*Lm*SodA	FM1	0.30	0.02	0.55	G_D-2_V-L_D-1_I	1.01	0.27	0.21
*S. pyogenes*	*Sp*SodA	FM1	0.38	0.16	0.58	A_D-2_V-L_D-1_I	0.99	0.37	0.39
*B. fragilis*	*Bf*SodFM2^d^	FM2	0.73	0.93^d^	0.27^d^	G_D-2_T-F_D-1_C^d^	24.7	0.86^d^	0.01^d^
*S. aureus*	*Sa*SodM	FM1	1.23	0.44	0.28	L_D-2_G-F_D-1_L	0.44	0.12	0.47
*A. muciniphila*	*Am*SodFM3^d^	FM3	4.03	0.69^d^	0.09^d^	…	…	…	…
c. *Wolfebacteria*	c*W*SodFM1^d^	FM1	18.5	0.47^d^	0.00^d^	V_D-2_G^d^	5.43	0.12^d^	0.00^d^
*E. coli*	*Ec*SodB	FM2	27.5	0.83	0.03	T_D-2_G-V_D-1_L	3.99	0.78	0.02
*N. gonorrhoeae*	*Ng*SodB	FM2	29.3	0.83	0.18	T_D-2_G	7.78	0.78	0.16
*B. anthracis*	*Ba*SodA2^c^	Fm1	Nd^c^	0.35	Nd^c^	…	…	…	…

Table summarizing the key data collected for all wild-type and mutated variants of the SodFMs under study herein. Data demonstrate each isozyme's metal preference (CR) and the determined redox state (nOS) as calculated from the UV-visible absorption spectra. Data presented here are a summary of those detailed in Source Data Table 1, which includes all individual results, errors, and statistical analyses, as well as the metal content measured within all isozyme preparations as determined by ICP-MS.

ND, not determined in this study.

^a^CR values were calculated by dividing the measured Fe-dependent activity by the measured Mn-dependent activity, as determined by a NBT/riboflavin-based spectrophotometric assay.

**Figure 1:**
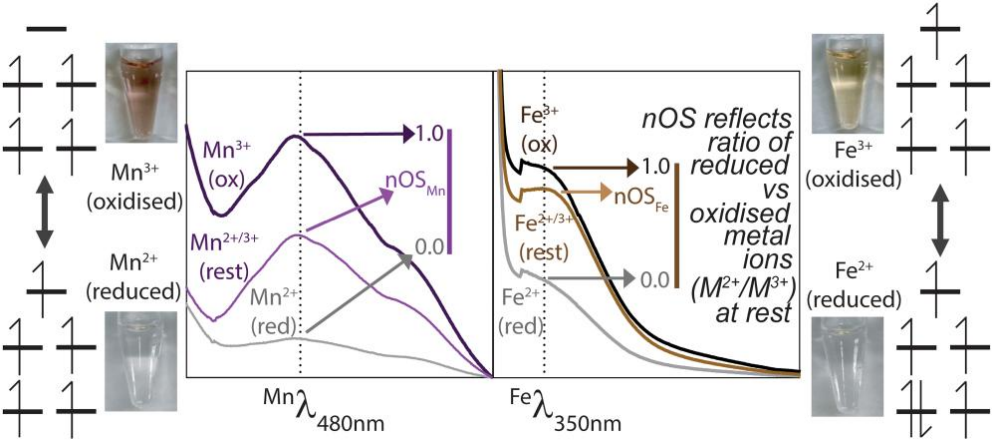
Schematic illustration of SodFM reaction, color and spectra, and calculation of nOS. UV-visible absorption spectra (central panels) of *Sa*SodA (left) and *Ec*SodB (right) loaded with Mn and Fe, respectively. The spectra of each protein as purified, which were at equilibrium under atmospheric aerobic conditions, are termed “resting spectra” and labeled M^2+^/M^3+^ (rest) and are shown with the lighter colored lines (Mn-*Sa*SodA, purple; Fe-*Ec*SodB, brown). Spectra of the oxidized protein samples are labeled M^3+^ (ox) and are shown in darker colored lines, and spectra of the reduced proteins are labeled M^2+^ (red) and are shown in gray. Spectra were obtained from samples containing approximately 100 µM protein in 20 mM Tris pH 7.5, 150 mM NaCl. Chemical oxidation was achieved by incubation with one mole equivalent of potassium permanganate and chemical reduction by incubation with three mole equivalents of sodium dithionite for 10 min, followed by extensive buffer exchange using centrifugal filtration into the same buffer. The derivation of nOS values by normalization of the resting spectral intensity, ^Mn^λ_480nm_ and ^Fe^λ_350nm_, against those of the fully oxidized and fully reduced samples is illustrated. Also shown (outer panels) are photographs of each oxidized (upper photo) and reduced (lower photo) protein samples, with their respective electronic configurations (high spin ions in a pseudo-trigonal bipyramidal configuration) schematically illustrated.

^b^Normalized oxidation state (nOS) values were calculated by normalizing the “resting” UV-visible absorption spectral peak intensity (^Mn^λ_480nm_ and ^Fe^λ_350nm_) between the fully reduced peak intensity (nOS = 0) and fully oxidized peak intensity (nOS = 1) (Fig. 1).

^c^Only one fully metal-loaded form of *Ec*SodA (in both its wild-type and mutated form) and *Ba*SodA2 could be produced, precluding measurement of activity of the other form, so CR values could not be calculated for these two isozymes.

^d^While all other SodFMs were analyzed in triplicate, *Bf*Sod, *Am*Sod, *Cw*Sod, and their mutant variants were analyzed as supplementary samples with *n* = 1 in order to test whether the trend, established from the core *n* = 3 dataset, was also observed in these more highly evolutionarily divergent isozymes.

Quantitative measurement of SodFM reduction potentials is challenging. The buried active site is only accessible via a narrow solvent channel, necessitating the use of small-molecule electrochemical mediators to communicate between the active site metal and an electrode, which is slow and inefficient and results in protein degradation over the course of experiments (Lawrence and Sawyer 1979; Verhagen et al. 1995; Lévêque et al. 2001). Given these limitations, it is not experimentally possible to measure reduction potentials of a diverse array of SodFMs and their mutated variants (Sendra et al. 2023) to explicitly test a role for redox tuning in SodFM evolution. Here, we leveraged the spectral features of their *d*-block metals, Fe and Mn, to assess the redox properties of SodFMs (Fig. 1). Both metals exhibit absorptions in the UV-visible region of the spectrum (Whittaker and Whittaker 1991; Vance and Miller 1998b). Mn^3+^ yields a broad, distinct feature from 400 to 700 nm with a maximum at 480 nm (defined herein ^Mn^λ_480nm_), whereas Fe^3+^ gives rise to a weak shoulder on the main polypeptide absorbance with a maximum at 350 nm (herein ^Fe^λ_350nm_). These features give rise to visible purple (Mn) or brown (Fe) colors in concentrated SodFM samples. Crucially, the intensities of these colors and the spectral features were increased after complete oxidation to Mn^3+^/Fe^3+^ and diminished on complete reduction to the Mn^2+^/Fe^2+^ oxidation state (Fig. 1). This indicates that the resting state, representing samples at aerobic equilibrium under standardized experimental conditions, contained mixtures of reduced and oxidized protein-bound metal cofactors. We thus utilized the peak intensity of these absorption features to assess the resting oxidation state of the active site metals in SodFM samples.

**Figure 2:**
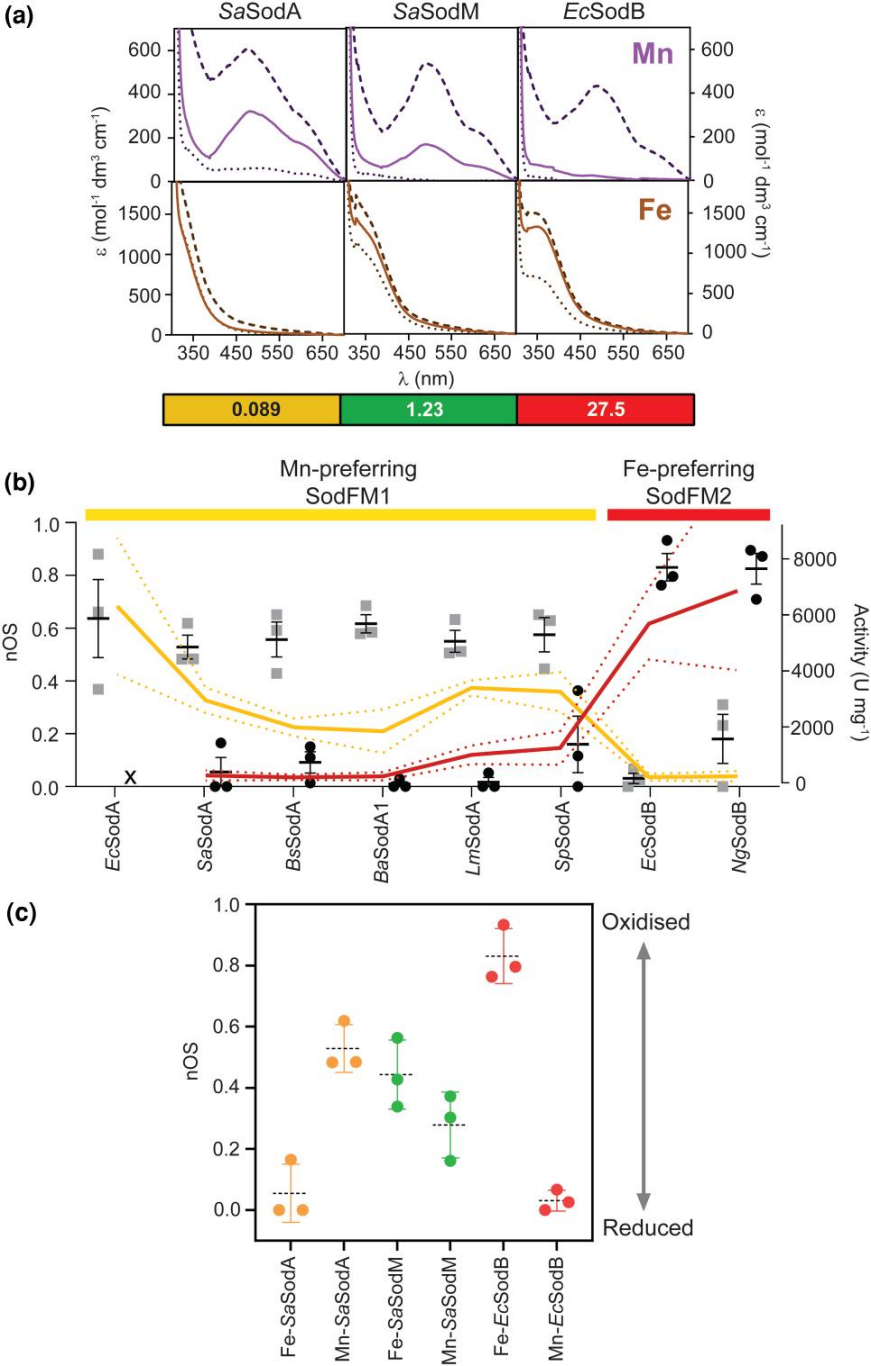
Spectra of SodFMs at rest correlate with their metal-dependent activity. a) UV-visible absorption spectra of Mn-loaded (upper panels, purple lines) and Fe-loaded (lower panels, brown lines) *Sa*SodA, *Sa*SodM, and *Ec*SodB. Resting spectra of proteins equilibrated under atmospheric conditions are shown as solid lines and spectra of oxidized and reduced proteins as dashed and dotted lines, respectively. The resting spectra lie between the respective oxidized and reduced spectra for catalytically active forms (Mn-*Sa*SodA, Fe-*Ec*SodB, Mn-*Sa*SodM, and Fe-*Sa*SodM) but are similar to reduced spectra for inactive forms (Fe-*Sa*SodA, Mn-*Ec*SodB). Spectra shown are *n* = 1 but representative of further replicates (*n* = 3). CR values are annotated below, colored by Mn preference (yellow), cambialistic (green), and Fe preference (red). b) Inverse trend between Mn-dependent (yellow line) and Fe-dependent activity (red line) detected for SodFM1 and SodFM2 isozymes and their normalized oxidation state (nOS) when loaded with Mn (gray bars) or Fe (black bars). The nOS values were calculated by normalizing resting spectral peak intensity (^Mn^λ_480nm_ and ^Fe^λ_350nm_) between those of reduced (nOS = 0) and oxidized (nOS = 1) peak intensity. Enzymes were ordered along the *x* axis according to their CR. All spectra and activities were measured in triplicate using independent biological replicates. Errors represent the standard deviation around the mean, displayed as error bars for nOS and dotted lines for activity in their respective colors. As we were unable to generate Fe-*Ec*SodA, no activity or nOS are displayed for this form (x indicates lack of data). c) Box and whisker plot comparing nOS from triplicate analyses of each SodFM metal form. Whiskers were calculated using Tukey's test (*n* = 3).

Initially, we sought to verify the relationship between the intensity of these spectral features and the metal's oxidation state using three well-studied model SodFMs that represent archetypes of the three traditional “metal specificities”: MnSOD (*S. aureus* SodA (Garcia et al. 2017), belonging to subfamily SodFM1), camSOD (*S. aureus* SodM (Garcia et al. 2017); SodFM1), and FeSOD (*E. coli* SodB (Yost and Fridovich 1973); SodFM2). We acquired resting, fully oxidized and fully reduced absorption spectra for each SodFM in both their Mn- and Fe-loaded forms under standardized experimental conditions (Fig. 2a). The resting ^Mn^λ_480nm_ was more intense for *Sa*SodA than for *Sa*SodM. This suggests that Mn-*Sa*SodA contained a higher proportion of oxidized Mn^3+^ cofactors. Conversely, the resting spectrum of catalytically inactive Mn-*Ec*SodB closely resembled that of its fully reduced form (Fig. 2a), resulting in ^Mn^λ_480nm_∼0, suggesting it contained almost exclusively reduced Mn^2+^ cofactors. The reverse was true for the Fe-loaded forms (^Fe^λ_350nm_: *Ec*SodB > *Sa*SodM > *Sa*SodA; Fig. 2a). We noted that the spectral intensities followed the trend in their metal-dependent catalytic activities (Table 1); those of enzymatically active forms were consistent with mixed oxidation state populations at rest, whereas inactive forms were more extensively reduced.

The absolute spectra varied between these three control SodFM isozymes, so peak spectral intensities were normalized to enable semiquantitative comparisons. Taking the spectra of the fully reduced and fully oxidized samples as representing the lower and upper bounds of absorbance, respectively, we generated normalized numerical values for ^Mn^λ_480nm_ and ^Fe^λ_350nm_ from each enzyme's resting spectrum (Fig. 1). The resulting values are taken to approximate the proportion of active site metal ions in the resting sample that are oxidized and are therefore termed normalized oxidation state (nOS), because nOS = 0 represents fully reduced and nOS = 1 represents fully oxidized samples. We found that all catalytically active forms of these SodFMs exhibited nOS values >0.25, indicating a significant proportion of oxidized metal cofactors at rest (Table 1). These nOS values are consistent with the ability to perform both the oxidative and reductive half-reactions of the SOD catalytic cycle under these conditions as the enzyme population at rest contains cofactors poised to perform either half-reaction. Conversely, the two SOD forms lacking activity, Fe-*Sa*SodA and Mn-*Ec*SodB, had nOS values close to zero, implying these samples contained exclusively reduced cofactors at rest (Table 1).

To validate these results, a larger panel of purified isozymes from the SodFM1 and SodFM2 subfamilies (Sendra et al. 2023), including enzymes from *Bacillus subtilis*, *Listeria monocytogenes*, *Streptococcus pyogenes*, and *Neisseria gonorrhoeae*, was analyzed (Figures S1 and S2). The spectra of these Mn-preferring SodFM1 (*Bs*SodA, *Lm*SodA, and *Sp*SodA) and Fe-preferring SodFM2 (*Ng*SodB) isozymes (Sendra et al. 2023) (Figure S3) were consistent with those of the canonical enzymes from *S. aureus* and *E. coli* (Fig. 2b). All the SodFM1s exhibited higher nOS_Mn_ values (0.53 to 0.64) but lower nOS_Fe_ (0.02 to 0.16), consistent with the strong Mn preference of their catalysis. The SodFM2 isozyme had a higher nOS_Fe_ (0.83) and a low nOS_Mn_ (0.03), consistent with its strong Fe preference (Table 1). Although we observed only minor variations in the wavelength of maximal absorbance (λ_max_) in the Mn spectra, which ranged between 472 and 485 nm, we saw greater variation in the maximal intensity (ε_max_) of the Mn spectra, which ranged from 109 (*Ng*SodB) to 606 M^−1^ cm^−1^ (*Sa*SodA), which might indicate distinct active site chemical environments around the manganese ion (Source Data Table 1).

Taken together, our data indicated that in SodFM1 and SodFM2, the two subfamilies most commonly found across the tree of life (Sendra et al. 2023), the enzyme's nOS values reflected their metal preference (Fig. 2c). When loaded with the wrong metal, the highly metal-preferring SODs had few oxidized cofactors at rest (nOS≈0), but were much more highly oxidized when loaded with their preferred cofactors. Notably, the cambialistic SOD had higher nOS values with both metals reflecting the presence of oxidized cofactors in both forms. These data suggested a trend between resting oxidation state and catalytic metal preference. Crucially, this trend was conserved across the SodFM1 and SodFM2 subfamilies that are derived from an ancient evolutionary split (Sendra et al. 2023), suggesting the hypothesis that evolutionary processes can shift catalytic metal preference by altering this biophysical property.

### Both metal preference and nOS are regulated by the SodFM secondary coordination sphere

**Figure 3:**
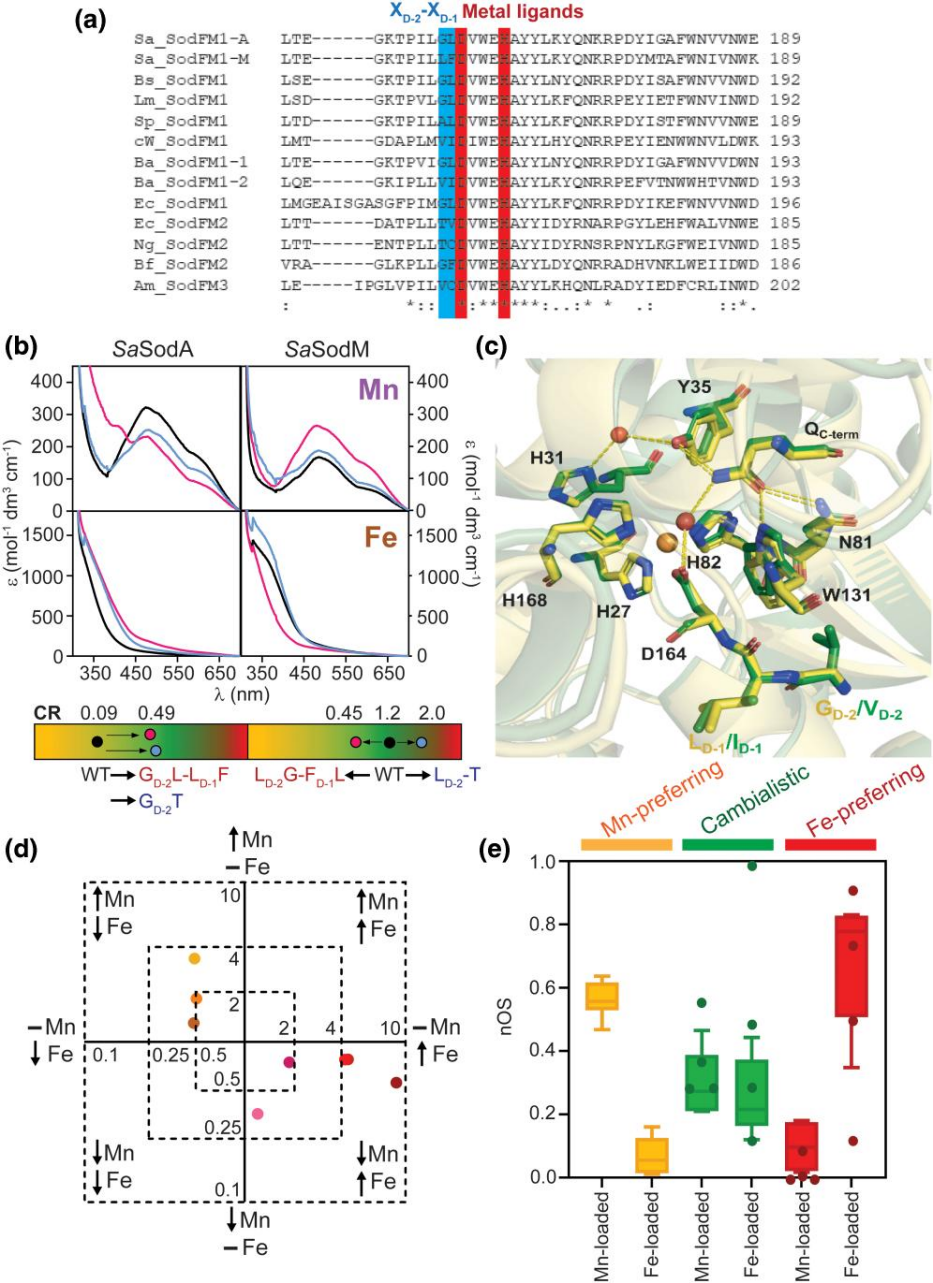
Spectra and nOS in mutated SodFM variants with altered metal preference. a) Section of a sequence alignment of all SodFMs in this study (Sa, *Staphylococcus aureus*; Bs, *Bacillus subtilis*; Lm, *Listeria monocytogenes*; Sp, *Streptococcus pyogenes*; cW, candidatus *Wolfebacteria*; Ba, *Bacillus anthracis*; Ec, *Escherichia coli*; Ng, *Neisseria gonorrhoeae*; Bf, *Bacteroides fragilis*; Am, *Akkermansia muciniphila*). Second-sphere residues targeted for mutagenesis, X_D-2_ (dark blue) and X_D-1_ (light blue), and two of the conserved metal ligands (red) are highlighted. Alignment was produced with Clustal Omega, and conservation levels are shown by symbols below the alignment where “*” represents fully conserved residues, “:” represents high, and “.” represents low level of conservation. b) UV-visible absorption resting spectra of *Sa*SodA (left) and *Sa*SodM (right) when Mn- (upper panels) and Fe-loaded (lower panels) for wild-type (black), X_D-2_/X_D-1_ (pink), and X_D-2_T (blue) mutant variants. Tricolor gradient panels below show the shift in CR from wild-type (black circles) to X_D-2_/X_D-1_ (pink circles) and X_D-2_T (blue circles) variants. Changes in spectral intensity were seen where mutagenesis caused a shift in metal preference. c) Ribbon and stick representation of superimposed (RMSD = 0.38 Å) active sites of *Lm*SodA (yellow; 1.65 Å) and the *Lm*SodA V_D-2_-I_D-1_ variant (green; 1.40 Å) displaying metal-coordinating ligands, water-coordinating glutamine, and X_D-2_X_D-1_ residues. d) Plot shows fold changes (log2 scale) in activity with Mn (orange) and Fe (red) of tested SodFM mutants relative to wild types (Table 1). Variants either gained Fe activity and lost Mn activity (*Sa*SodA, red; *Bs*SodA, burgundy, *Ba*SodA1, dark red; *Lm*SodA, light pink; *Sp*SodA, dark pink) or gained Mn activity and lost Fe activity (*Sa*SodM, brown; *Ec*SodB, yellow; *Ng*SodB, orange). e) Box and whisker plot of nOS from replicated wild type and mutant SodFM analyses, grouped by metal preference. Whiskers calculated using Tukey's test (*n* = 3), nOS were calculated from spectra as previously described. Overlaid points display nOS of divergent wild-type and mutant SodFMs analyzed in single replicate (*n* = 1).

Numerous studies have demonstrated the role in determining SodFM metal preference played by residues localized within the metal cofactor's secondary coordination sphere (Whittaker and Whittaker 1997; Hiraoka et al. 2000; Schwartz et al. 2000; Yamakura et al. 2003; Miller 2008; Hunter et al. 2018; Barwinska-Sendra et al. 2020; Sendra et al. 2023). These residues have been altered during evolutionary adaptations that have resulted in changes of metal preference of SodFM catalysis (Barwinska-Sendra et al. 2020; Sendra et al. 2023). We hypothesized that metal-preference changes caused by mutations in these residues would also be associated with changes in nOS. To test this, we calculated nOS values for a set of SodFM variants whose metal preference had been altered through mutation of second-sphere residues (Fig. 3a). Mutations were designed to mimic observed evolutionary events of metal-preference modulation (Sendra et al. 2023), by switching native residues for those observed in the same locus in other SodFM isozymes that have contrasting metal preferences.

We previously demonstrated the residue X_D-2_ was key to the evolutionary divergence of the staphylococcal SodFM1s (Barwinska-Sendra et al. 2020). Its neighboring residue, X_D-1_, plays a moderating role in metal preference (Barwinska-Sendra et al. 2020). X_D-2_ was also shown to be important in the evolution of metal preference in other SodFMs sampled across the tree of life (Sendra et al. 2023). Reciprocal mutagenesis of this amino acid pair in the *S. aureus* SodFMs (G_D-2_-L_D-1_ in Mn-preferring *Sa*SodA, CR = 0.089; L_D-2_-F_D-1_ in cambialistic *Sa*SodM, CR = 1.225) substantially inverted their metal preferences as well as inverting the susceptibility of their Mn^3+^ forms to chemical reduction by dithionite (Barwinska-Sendra et al. 2020), consistent with these residues modulating redox properties of the metal. The *S. aureus* SodFMs are a model of metal-preference evolution because both their physiological role during infection (Garcia et al. 2017) and their biochemical and structural properties (Barwinska-Sendra et al. 2018, 2020) were previously established. Here, we first confirmed that mutagenesis of X_D-2_/X_D-1_ created a more cambialistic variant of *Sa*SodA (L_D-2_-F_D-1_, CR = 0.490) and a more Mn-preferring variant of *Sa*SodM (G_D-2_-L_D-1_, CR = 0.448) (Table 1). Variants of both *Sa*SODs were also created with the non-native substitution T_D-2_, a residue common in isozymes of subfamily SodFM2 (Sendra et al. 2023) that is most commonly indicative of Fe-preferring SODs (Vance and Miller 2001; Wintjens et al. 2004; Miller 2008; Sendra et al. 2023). Indeed, this mutation increased Fe-dependent activity in both cases (*Sa*SodA T_D-2_, CR = 0.594; *Sa*SodM T_D-2_, CR = 1.991) (Table 1). Crucially, all of these mutations also affected the resting spectra (Fig. 3b) and the resulting nOS values (Table 1). The variants *Sa*SodA-L_D-2_-F_D-1_, *Sa*SodA-T_D-2_, and *Sa*SodM-T_D-2_, all of which exhibited increased catalysis with Fe, also showed elevated nOS_Fe_. The *Sa*SodA-L_D-2_-F_D-1_ variant exhibited significantly decreased nOS_Mn_, consistent with its reduced Mn activity. The *Sa*SodM G_D-2_-L_D-1_ variant, which had increased Mn preference, exhibited the opposite trend of increased nOS_Mn_ and significantly decreased nOS_Fe_.

After T_D-2_, the amino acid most frequently found at this position in Fe-preferring SodFM enzymes is valine (Sendra et al. 2023). We therefore introduced V_D-2_ mutations into each of the Mn-preferring enzymes, *Bs*SodA, *Lm*SodA, and *Sp*SodA (CR = 0.134, 0.296, and 0.384, respectively), by switching their X_D-2_/X_D-1_ residues for those of the Fe-dependent (Tu et al. 2012) (Table 1) SodFM1 *Ba*SodA2 (V_D-2_-I_D-1_). In all cases, the variants exhibited increased Fe preference (CR = 1.487, 1.014, and 0.987, respectively; Table 1). These mutated *Bs*SodA, *Lm*SodA, and *Sp*SodA variants also showed changes in spectra and nOS (Table 1), increasing nOS_Fe_ and decreasing nOS_Mn_, consistent with their cambialistic character.

X-ray crystallographic structural analysis of *Lm*SodA and *Ng*SodA demonstrated the anticipated architecture of their active sites (Figure S4). Crucially, comparison of the structure of WT and the V_D-2_-I_D-1_ variant *Lm*SodA structures showed that their active site and hydrogen-bonding networks superimposed identically within the limits of crystallographic resolution (Fig. 3c), consistent with previous studies of the *Sa*SODs and their variants (Barwinska-Sendra et al. 2020; Sendra et al. 2023). These data limit a role for substantial rearrangement of the active site in explaining the distinct metal preferences caused by these mutations. Instead, this is consistent with secondary coordination sphere residues tuning the redox properties of the metal ion through a mechanism not visible *via* X-ray crystallography with currently available resolution.

Finally, we constructed an X_D-2_/X_D-1_ mutant of the Fe-preferring SodFM2 isozyme, *Ng*SodB (T_D-2_-C_D-1_, CR = 29.285), swapping them for the equivalent residues from the cambialistic (Sendra et al. 2023) SodFM2 from *Bacteroides fragilis* (*Bf*SodFM2 G_D-2_-F_D-1_, CR = 0.728; Table 1). The *Ng*SodB G_D-2_-F_D-1_ was inactive and poorly expressed, in contrast with the active G_D-2_ single mutant, suggesting the amino acid at X_D-1_ can stabilize the presence of certain residues at the X_D-2_ position (Barwinska-Sendra et al. 2020). Despite a reduction in Fe activity and CR, *Ng*SodB G_D-2_ remained a highly Fe-preferring enzyme (CR = 7.783). Its nOS values also showed only minor, insignificant changes with respect to the wild type consistent with this slightly modulated metal preference (Table 1).

Importantly, we observed that our variants either gained Fe activity and lost Mn activity or gained Mn activity and lost Fe activity, indicating an inverse relationship between these two activities (Fig. 3d). The trend of simultaneous change in nOS and CR of these variants with mutations in the key X_D-2_ position that altered their metal preference (Fig. 3e) matched the pattern that was observed in comparisons of wild type variants (Fig. 2c). Together, these data are consistent with the hypothesis that metal-preference changes are dependent on modulation of metal cofactor resting redox state, as measured by nOS.

### Role of classical neofunctionalization in the emergence of cambialistic SodM in *S. aureus*

**Figure 4:**
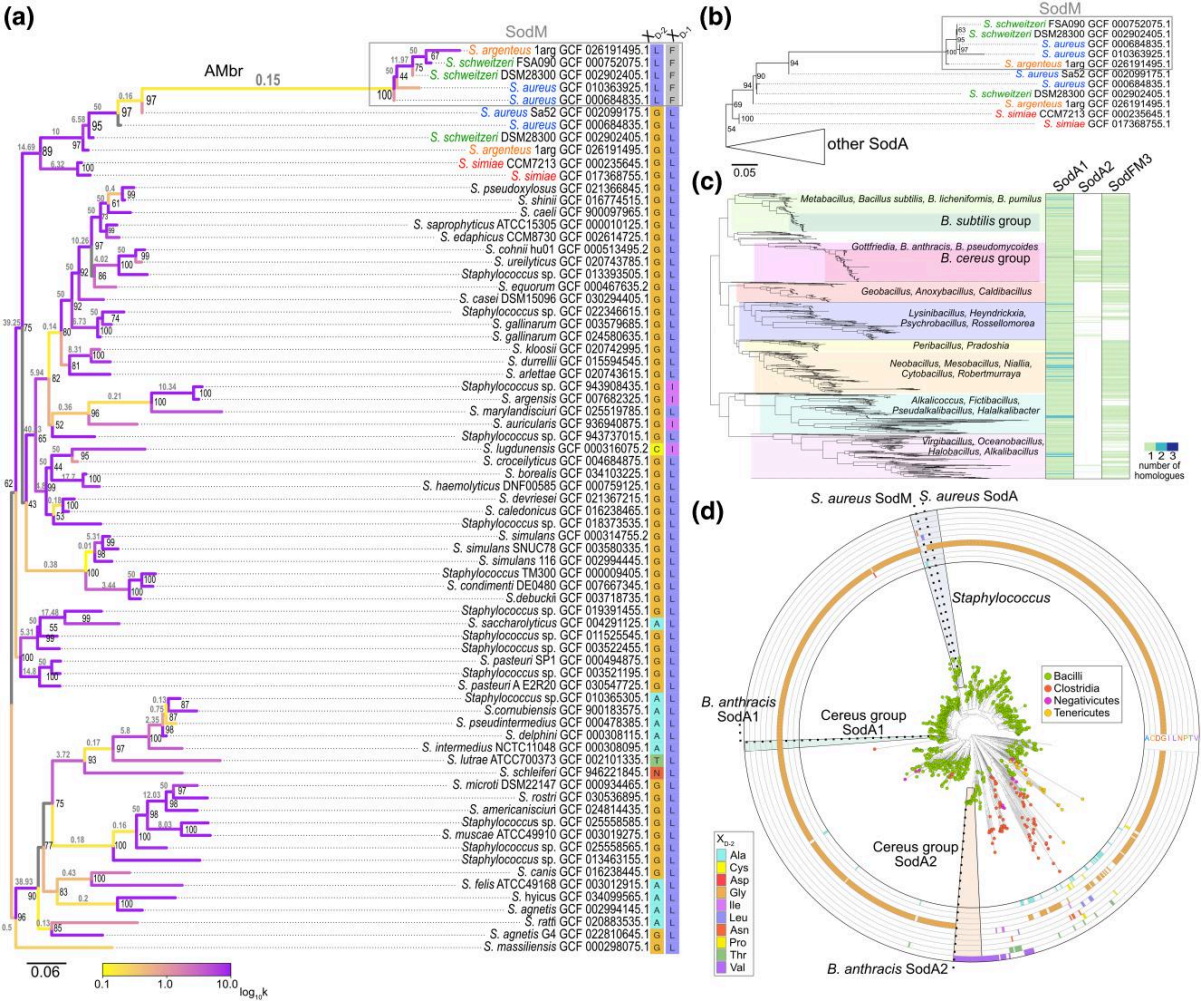
Investigating evolutionary mechanisms involved in SodFM1 metal-preference modulation. a) Maximum likelihood (GTR + F + R4 model) phylogeny of representative *sodFM1* nucleotide sequences sharing less than 98% identity sampled from all 21,452 available *Staphylococcus* genomes. All *sodM* sequences (black rectangle) from *S. aureus* (green), *S. argenteus* (orange), and *S. schweitzeri* grouped together with *sodA* sequences from the same species. Sequences of *sodA* from their closest relative (*S. simiae*, red) formed an outgroup, consistent with vertical inheritance of *sodA*. Branches of the tree were color-coded with branch-dependent selection intensity parameter (*k*, gray values above branches). Values of *k* < 1 (yellow) indicate relaxed selection, and those of *k* > 1 (purple) indicate intensified selection. b) Maximum likelihood (LG + G4) protein tree displaying topology consistent with that of the nucleotide tree (A). Support values (black, A and B) correspond to ultrafast bootstrap values from 1,000 replicates. Scale bars represent the number of substitutions per site. c) Species tree of 1,115 nonredundant *Bacillaceae* genomes. Number of identified SodFM1 (SodA1 and SodA2) and SodFM3 homologs were mapped onto the tree (blue-green heat map). d) Protein tree of SodFM1s sampled from across 828 representative Firmicutes and all available 11,394 *Bacillaceae* genomes. The identity of the X_D-2_ residues was mapped in the concentric circles. *S. aureus* SodM grouped together with SodA homologs, providing evidence for its emergence from a duplicated SodA ancestor. *B. anthracis* SodA2 grouped more closely with SodFM1s from Clostridia than *B. anthracis* SodA1 suggesting possible acquisition *via* LGT.

We previously hypothesized that cambialistic *S. aureus* SodM had most likely emerged via neofunctionalization from its Mn-preferring SodA ancestor (Barwinska-Sendra et al. 2020), enabling *S. aureus* to resist Mn starvation during infection (Garcia et al. 2017). However, the exact evolutionary mechanism of this process remained unexplored. In our previous analysis of SodFMs sampled across the tree of life (Barwinska-Sendra et al. 2020), SodM homologs, found only in the *S. aureus/S. argenteus* lineage, grouped at the base of SodA sequences from all oxidase-negative *Staphylococcus*, rather than with only SodA sequences from *S. aureus/S. argenteus*, as would be expected if they originated *via* gene duplication. Therefore, we repeated the analyses using 73 nonredundant (<98% pairwise nucleotide sequence identity) SodFM1s identified in all 21,452 available *Staphylococcaceae* genome assemblies. With this improved sampling, SodM and SodA from the *S. aureus/S. argenteus* lineage grouped together in both nucleotide (Fig. 4a) and protein (Fig. 4b) phylogenies with high support (97% and 94% bootstrap support values, respectively), consistent with SodA and SodM emerging from ancestral gene duplication.

Next, we tested for evidence of the relaxation of selection following the inferred ancestral *sodA* duplication (Wertheim et al. 2015). When SodM branches were tested against the background of all SodA, inferred relaxation (*K* = 0.61) was significant (*P* = 0.000, likelihood ratio = 14.49). Mapping of the branch-dependent selection intensity parameter (*k*, Fig. 4a) onto the SodA/M phylogeny was consistent with *S. aureus/S. argenteus/S. schweitzeri* SodM and SodA being under intensified selection (*k* > 1), while evidence of relaxed selection was mainly found on the long branch connecting SodAs and SodMs (AMbr, *k* = 0.15). In an analysis excluding the AMbr, inferred selection intensification (*K* = 2.08) was not significant (*P* = 0.226, LR = 1.46). This provided evidence in support of selection relaxation along the AMbr branch during the emergence of the last common SodM ancestor but not among the extant SodMs. Based on our biochemical and sequence analyses, position X_D-2_ clearly played a role in the evolution of cambialism in SodM (Barwinska-Sendra et al. 2020; Sendra et al. 2023). Although they revealed evidence of episodic diversifying selection (*P* = 0.000; BUSTED), neither of our analyses (*P*-value = 0.67; MEME) provided any evidence for it acting on the residue X_D-2_. However, we did detect evidence for X_D-2_ being under pervasive negative selection (posterior probability [α > β] = 0.937; FUBAR) alongside 170 of 191 tested sites, consistent with a conserved role for both SodA and SodM in extant *S. aureus*. Previously, we demonstrated that multiple different single mutations at this site can change biochemical properties of SodM toward higher Mn or Fe preference (Sendra et al. 2023). Therefore, negative selection acting at this site is consistent with our previous hypothesis that it was not simply higher Fe preference but rather cambialism that was selected for in SodM (Sendra et al. 2023). Altogether, our results suggest that SodM evolved during a period of relaxed selection following the inferred duplication of the ancestral *sodA*, followed by intensified selection of its neofunctionalized descendent *sodM*, with likely retention of its cambialism through purifying selection.

According to the classical neofunctionalization model (Ohno 1970), following a gene duplication, one copy remains under purifying selection, while a second copy experiences a period of relaxed selection, which can facilitate acquisition of new properties that can be subsequently fixed. This classical model has been criticized and alternative models postulated (Hughes 1994; Force et al. 1999; Bergthorsson et al. 2007). However, the following empirical observations are consistent with SodM evolution having occurred according to the classical model: (i) although the ancestral capacity to function with Mn is diminished in cambialistic *Sa*SodM, it was never completely lost (Garcia et al. 2017; Barwinska-Sendra et al. 2020; Sendra et al. 2023); (ii) SodM is capable of forming functional heterodimers with *Sa*SodA (Garcia et al. 2017); and (iii) expression of *sodM* and *sodA* in *S. aureus* is differentially regulated (Garcia et al. 2017), which, if acquired early, could have enabled the two copies of the duplicated gene to experience different selection intensities. Taken together with our prior study (Barwinska-Sendra et al. 2020), this analysis establishes a likely evolutionary pathway for biological switching of catalytic metal preference in a natural SodFM.

### Modulation of nOS underlies evolutionary changes of metal preference in natural SodFMs

Our data demonstrated that the SodFM resting spectral features and their resulting nOS values were predictive of the metal preference of natural SodFMs and that secondary sphere mutations that altered SodFM metal preference concomitantly altered nOS. The most recent identified evolutionary change in SodFM metal preference, the neofunctionalization of the SodFM1 *Sa*SodM following duplication of the ancestor of Mn-preferring *Sa*SodA (Barwinska-Sendra et al. 2020), was associated with a change in nOS_Fe_ (Fig. 2). Next, we tested whether the modulation of nOS has played a conserved role in multiple other evolutionary metal-preference switches that we have inferred from our analyses of natural enzymes sampled across the tree of life (Sendra et al. 2023).

The only SodFM1s previously shown to be Fe-preferring are *B. anthracis* SodA2 (Tu et al. 2012) and the SodFM1 from candidatus *Wolfebacteria* (Sendra et al. 2023) (c*W*SodFM1; CR = 18.48) of the candidate phyla radiation (CPR). The CPR are a large and diverse phylum of bacteria that are poorly characterized and mostly uncultured, known primarily through their metagenome sequences. Like *Ba*SodA2, the c*W*SodFM1 enzyme also evolved from a Mn-preferring ancestor but through a different molecular pathway which also included change of the catalytic water-coordinating residue from Gln to His (Sendra et al. 2023). The c*W*SodFM1 also displayed nOS values consistent with its cofactor preference (Table 1). We constructed a G_D-2_ mutant of c*W*SodFM1, converting the valine residue in this position to a glycine, which is more typical of SodFM1 and generally indicative of Mn preference (Sendra et al. 2023). This variant exhibited diminished Fe activity without increased Mn activity (c*W*SodFM1 V_D-2_G, CR = 5.432) with a corresponding decrease in nOS_Fe_ (Table 1). Thus, isozymes from the predominantly Mn-preferring SodFM1 subfamily in which independent evolutionary alterations in metal preference have been observed, creating either a cambialistic (*Sa*SodM) or an Fe-preferring enzyme (*Ba*SodA2 and c*W*SodFM1), exhibited consistent alterations in their spectral properties and nOS.

Next, we tested whether altered nOS was associated with changes in metal preference in other SodFM subfamilies. We characterized an atypical cambialistic (Sendra et al. 2023) (CR = 0.728) *B. fragilis* isozyme (*Bf*SodFM2) from the predominantly Fe-preferring subfamily SodFM2. Both metal forms of *Bf*SodFM2 had high nOS values, consistent with its cambialistic activity (Table 1), analogous with cambialistic *Sa*SodM. We also created the T_D-2_-C_D-1_ variant of *Bf*SodFM2 in which we introduced residues from Fe-preferring SodFM2 *Ng*SodB. This mutation reverted *Bf*SodFM2 to its most likely ancestral Fe-preferring state, retaining high Fe activity (CR = 24.67) and nOS_Fe_ while strongly diminishing Mn activity and nOS_Mn_ (Table 1).

Finally, we investigated a member of the SodFM3 subfamily. Isozymes from SodFM3 and SodFM4 have a more distant sequence, structural, and phylogenetic relationship (Sendra et al. 2023) with the isozymes from SodFM1 and SodM2. Nonetheless, we observed nOS values for the Fe-preferring (CR = 4.027) SodFM3 from *Akkermansia muciniphila* (*Am*SodFM3) (Sendra et al. 2023) that were consistent with those of the characterized Fe-preferring SodFM1 and SodFM2 enzymes (Table 1). This showed that the redox properties of these tetrameric SodFMs display characteristics similar to those observed in the dimeric enzymes of the SodFM1 and SodFM2 subfamilies.

The trend of nOS following metal preference of activity was reproduced across the entire dataset (Fig. 3e). Collectively, these data demonstrate that all the examples of inferred evolutionary modulation of SOD metal preference identified so far (Barwinska-Sendra et al. 2020; Sendra et al. 2023), including the ancient split between the SodFM subfamilies, and the more recent changes in metal preference found in *S. aureus*, the bacilli, the *Bacteroides*, and the CPR bacteria, all resulted in concomitant changes in nOS. These observations are consistent with the hypothesis that alterations in the redox properties of the active site apparent as changes in nOS play a conserved role in the evolutionary modulation of SOD metal preferences.

### Unexpected metal-binding by two SodFM1s with opposite metal dependence

A pair of SodFM1s with different metal preferences in *B. anthracis* (*Ba*SodA1 and *Ba*SodA2) (Tu et al. 2012) bears cursory resemblance to the pair identified in the staphylococci. We tested whether the SodA2 enzyme was acquired in an evolutionary process analogous to that described in *S. aureus* (Barwinska-Sendra et al. 2020). As previous data suggested that *Ba*SodA2 is highly Fe-preferring (Tu et al. 2012), it is unlikely that it evolved in a process identical to that of *Sa*SodM as the complete loss of the ancestral Mn activity could have posed challenges in maintaining the duplicated gene in the population. The scattered distribution of the SodA2 homologs found across the *Bacillaceae* species tree (Fig. 4c) is suggestive of multiple independent lateral gene transfer (LGT) events. The original source of the transferred genes is unclear, but the protein tree of SodFMs sampled across *Firmicutes* (Fig. 4d) suggests that SodA2s may have originated from outside *Bacillaceae*. Grouping of the SodA2s with their closest *Clostridiaceae* homologs is nonetheless weakly supported (38% bootstrap value), reflecting their high level of sequence divergence. Therefore, the most parsimonious explanation is the acquisition of SodA2 via LGT in the ancestor of *B. anthracis/B. cereus*. However, the alternative hypothesis of emergence from the *Bacillus* SodA1 ancestor, followed by extreme sequence divergence that masks the phylogenetic signatures of SodA2's true origin, cannot be fully excluded. Regardless of the mechanism, the result is a distinct SodFM1 repertoire in extant pathogenic species of the *Bacillus cereus*/*anthracis* group, including many pathogens, compared with their relatives including those from the lineage of nonpathogenic *B. subtilis* (Fig. 4c). Consistent with its high protein sequence homology to *Sa*SodA and *Bs*SodA (66% and 77% identical, respectively), *Ba*SodA1 shows canonical Mn preference (Table 1) and likely reflects the state inherited from the last common *Bacillales* ancestor. However, while neofunctionalization of *Sa*SodM created a cambialistic enzyme (Barwinska-Sendra et al. 2020), *Ba*SodA2 has undergone a more extreme shift in metal preference toward Fe (Table 1). Crucially, we observed metal-binding selectivity in *Ba*SodA2, as its Mn-loaded form could not be generated under our standard heterologous expression conditions or even using a range of in vitro unfolding/refolding dialysis protocols (Tu et al. 2012) (Figure S5). This precluded assessment of its nOS_Mn_ and its Mn-dependent catalytic activity and thus CR, but was consistent with a previous study that demonstrated *Ba*SodA2 was associated exclusively with Fe in its native cytosol (Tu et al. 2012). Importantly, while *Ba*SodA1 exhibited typical spectra and nOS values expected from a Mn-preferring SodFM1, *Ba*SodA2 exhibited nOS_Fe_ = 0.35, consistent with its characterization as an Fe-active enzyme (Figure S3; Table 1). These atypical properties reflect the high level of SodA2 sequence divergence and can potentially explain the difficulty in inferring its origins. Furthermore, they may also explain the relatively high frequency of sharing SodA2s via LGT in *Bacillaceae*, as opposed to, for example, acquisition of more common Fe-preferring SodFM2s or SodFM3s. It is possible that selectivity for Fe binding may be more difficult to achieve in SodFMs and that it can provide some unique selective advantages. These data also pose a question about the evolutionary mechanism that could lead to emergence of an Fe-selective enzyme from a highly Mn-preferring ancestor and why such selectivity is not more widely observed among strongly metal-preferring SodFMs. Neofunctionalization cannot be completely excluded as we previously observed a complete switch between Fe preference and Mn preference *via* a single mutation in *Wosearchaeota* SodFM4 (V_D-2_ to A_D-2_ mutation) (Sendra et al. 2023). However, no such mutations are known to be possible in Mn-SodFM1s thus far. Therefore, given our current knowledge, subfunctionalization seems a more likely mechanism, suggesting the existence of at least one intermediate cambialistic ancestor of SodA2 prior to the emergence of Fe specificity and/or Fe selectivity.

Reciprocal double mutagenesis at X_D-2_/X_D-1_ of the *Ba*SodFM1s was performed (G_D-2_-L_D-1_ in Mn-preferring *Ba*SodA1, CR = 0.196; V_D-2_-I_D-1_ in Fe-active *Ba*SodA2, CR = unmeasurable) to investigate if these residues can play a role in switching metal preference in the bacilli, as in the staphylococci. The shift in metal preference toward cambialism in the *Ba*SodA1 V_D-2_-I_D-1_ mutant (CR = 0.743) was analogous to that observed in mutagenesis of *Sa*SodA, as were its significant changes in nOS (Table 1). The reciprocal mutation, inserting the G_D-2_-L_D-1_ residues of *Ba*SodA1, which are typical of SodFM1s and generally indicate Mn-preference (Sendra et al. 2023), into *Ba*SodA2 created an unstable and inactive variant. A G_D-2_ single mutant remained Fe-active when analyzed by in-gel assay from cell lysates; however, neither variant could be purified in sufficient quantity to analyze their spectra. Therefore, we conclude that the challenging adaptation to Fe activity in this SodFM1 has made its active site less able to switch between metal preferences through this mutagenesis strategy. This is consistent with our previous data for c*W*SodFM where a second mutation of water-coordinating His to Gln was required to increase its Mn-dependent activity and for the SodFM2 from *Agrobacterium tumefaciens* that we were unable to switch to its most likely ancestral Mn preference (Sendra et al. 2023). This suggests the presence of secondary epistatic mutations in SodFMs subjected to the most extreme evolutionary changes is required to induce metal-preference modulation (*At*SodFM2, *cW*SodFM1, *Ba*SodA2) and to cause metal selectivity (*Ba*SodA2) while retaining specific activity and protein fold stability.

Notably, *Ba*SodA2 was not the only isozyme that displayed unanticipated metal-binding during recombinant production. Despite its widespread adoption as a “model” Mn-preferring SodFM1 for decades (Whittaker and Whittaker 1997; Vance and Miller 2001; Whittaker et al. 2006), we observed here, as previously (Sendra et al. 2023), that *Ec*SodA has unusual properties. Under our standardized expression conditions in minimal medium, essential to control metal-loading of SodFM isozymes, *Ec*SodA was the only SodFM tested that did not bind Fe inside *E. coli* cells cultured aerobically in the absence of Mn. Under these conditions, *Ec*SodA was expressed only at low levels, and resulting protein preparations lacked significant Fe (Sendra et al. 2023). This observation is particularly striking as we were able to produce multiple SodFMs from all subfamilies sampled from across the tree of life in the same system (Sendra et al. 2023), but it was the homologously expressed *E. coli* SodA that displayed unusual properties, suggesting that it may represent a specific adaptation. Synthesis of the Fe form of *Ec*SodA required the use of a rich growth medium supplemented with additional Fe. Under these conditions, the metal-loading of the isozyme could not be fully controlled and was loaded with mixtures of Fe and Mn, which only allowed a limited spectral analysis to be performed. Crucially, a preparation of *Ec*SodA that contained mostly (∼80%) Fe exhibited a spectrum consistent with the other characterized Mn-preferring SodFM1s (Figure S3).

We reciprocally interconverted the two second-sphere residues in the *E. coli* SodFM1/SodFM2 pair (G_D-2_-L_D-1_ in Mn-preferring *Ec*SodA, CR = unmeasurable; T_D-2_-V_D-1_ in Fe-preferring *Ec*SodB, CR = 27.51). *Ec*SodB G_D-2_-L_D-1_ showed reduced Fe activity and a CR shift toward cambialism but remained Fe-preferring (CR = 3.992) and consequently exhibited only small changes in nOS that were not significant (Table 1). *Ec*SodA T_D-2_-V_D-1_ was still a highly Mn-active enzyme (∼70% of wild-type activity) with a high nOS_Mn_ (Table 1). However, like the wild type, the Fe-loaded form of *Ec*SodA T_D-2_-V_D-1_ could not be generated under our standard conditions in sufficient yield for spectroscopic analysis, suggesting different sites are responsible for its unusual metal-binding properties. Taken together, the *E. coli* SodFMs, unlike many of their SodFM1 (*Ec*SodA) and SodFM2 (*Ec*SodB) relatives, display a high degree of resistance to metal-preference alteration *via* the common metal-preference modulation pathways involving mutation of the X_D-2_-X_D-1_ residues (Sendra et al. 2023).

In summary, two SodFMs from *E. coli* and *B. anthracis*, uniquely among all the isozymes we have biochemically tested, exhibited a degree of metal selectivity under the tested conditions. Whereas all other SodFMs are competent to bind both metal cofactors, regardless of which confers catalysis, these enzymes displayed binding to only one of the respective metals, Mn or Fe, when expressed inside aerobically grown Δ*sodA*Δ*sodB E. coli* cells under defined metal culture conditions. Notably, SodA is already present within the manganese-poor cytosol of *E. coli* prior to an oxidative stress response (Anjem et al. 2009), and emergence of a level of metal selectivity might prevent its mis-metalation. If so, a need for *B. anthracis* to have *Ba*SodA2 already present in its manganese-rich cytosol (Helmann 2014) could also be hypothesized. Remarkably, two distinct isozymes with a degree of metal selectivity have evolved within the SodFM1 subfamily from most likely Mn-preferring, metal-nonselective ancestors, one with retention of the ancestral metal preference (Mn-selective *Ec*SodA) and the other with a metal-preference switch (Fe-selective *Ba*SodA2). These enzymes could prove to be useful models to study the mechanisms of metal-binding in SodFMs.

### The nOS reflects the enzymatic activity and resting redox poise of SodFM cofactors

Comparison of the nOS values derived from spectral analyses of ten wild-type isozymes and nine mutated variants illustrated an intriguing trend (Fig. 3e). Metalated forms of all SodFMs that exhibited significant catalytic activity also possessed substantial nOS values (generally 0.25 < nOS < 0.95). This indicates that all of these catalytically active samples contained a significant population of oxidized metal cofactors at rest. Conversely, all forms of SodFMs that exhibited negligible catalytic activity possessed nOS values close to zero, consistent with their metal ions being completely reduced at rest.

**Figure 5:**
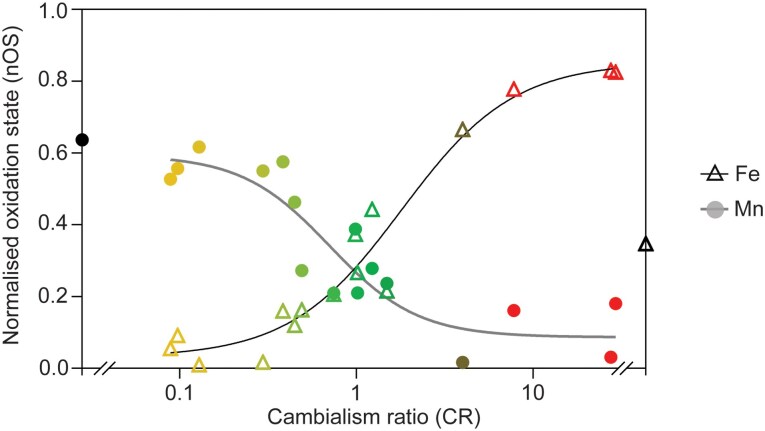
Inverse trends of CR and oxidation state in Mn-loaded and Fe-loaded SodFMs. When nOS values for Fe-loaded (open triangles) and Mn-loaded (closed circles) forms of all the SodFM isozymes analyzed in triplicate were plotted against CR on a logarithmic scale, we detected two inverse trends. The enzymes with low activity have very low oxidation at rest, while those with increased activity displayed higher oxidation at rest, for both metal forms. Data points are colored by CR using a tricolor gradient from yellow (Mn-preferring) to green (cambialistic) to red (Fe-preferring). Trendlines were calculated in GraphPad Prism (Version 9.3.1) using a sigmoidal four-parameter logistic model. The *R*^2^ values of the nonlinear fit to the data were *R*^2^ = 0.957 for the Fe-loaded enzymes and *R*^2^ = 0.841 for the Mn-loaded enzymes. As a real CR could not be calculated for *Ba*SodA2 due to apparent metal-discriminating selectivity of binding, this has been artificially assigned to the extreme of the CR scale, with its single nOS value represented by a black symbol, which was not used in the calculation of trendlines.

To determine the relationship between metal preference and nOS for each metal cofactor, we plotted each set of nOS values, nOS_Fe_ and nOS_Mn_, from our replicated spectral analyses of SODs against their CR (plotted on a logarithmic scale; Fig. 5). This clearly illustrated that metal preference is a continuum between perfect metal specificity extremes (here represented by the more metal-selective *Ec*SodA and *Ba*SodA2, whose nOS values could not be fully assessed), rather than the traditional view of three discrete states (MnSOD, FeSOD, camSOD). Trendline fitting of these datasets yielded a striking observation; nOS_Fe_ is proportional, while nOS_Mn_ is inversely proportional to CR (Fig. 5). The resulting trendlines intersected at CR = 0.97 and nOS = 0.27, representing the anticipated properties of a hypothetical, near-perfectly cambialistic isozyme. Furthermore, this trend still broadly held when additional data, acquired from the more highly divergent SodFMs and their mutants (Fig. 3e), were included (Figure S6).

Taken together, the trend observed between CR and nOS of all tested isozymes is strongly indicative that they are intrinsically linked features of SodFMs. The diverse SodFMs analyzed here represent a spectrum of evolutionarily divergent Fe/Mn SODs and include examples shown to have undergone ancient or more recent evolutionary modulations in preference (Sendra et al. 2023). This argues that this relationship between the metal preference and the cofactor's resting redox properties underlies the evolutionary mechanism of metal-preference switching. We propose that this relationship is determined by the effects of the protein architecture on the biophysical properties of the metal cofactor within this metalloenzyme family, mediated by the ion's secondary coordination sphere, and that evolution must alter nOS in order to modulate metal preference.

## Discussion

Metalloproteins are ubiquitous in biology. Metals have essential catalytic or structural functions in enzymes from all six Enzyme Commission classes. Metalloenzymes comprise nearly half of all oxidoreductases, in which *d*-block metals confer redox activity (Waldron et al. 2009). It is generally assumed that such redox metalloenzymes are highly specific for their cognate metal (Foster et al. 2014; Imlay 2014; Valdez et al. 2014) because they optimize their active site to manipulate the metal's reduction potential to achieve a suitable midpoint potential for efficient catalytic cycling. This optimization is called redox tuning. Due to inherent differences in electronic configurations of different metals, the tuning imparted by a protein structure on its cognate metal ion (e.g. Fe) will be inappropriate for tuning the distinct potential of a non-cognate metal cofactor (e.g. Mn). The redox tuning model can explain why there is only a few known natural metalloenzymes that exhibit cofactor flexibility (cambialism), although recent analyses suggest they may be more common in nature than previously thought (Dai et al. 1999; Emerson et al. 2008; Valdez et al. 2014; Garcia et al. 2017; Sendra et al. 2023; Liu et al. 2024). When a metalloenzyme that is optimized for one redox metal cofactor evolves an alternative metal specificity, its active site must be re-optimized for the new metal. This process could enable the new metalloenzyme to gain an entirely new catalytic function using the new cofactor, an idea supported by numerous bioinformatic observations in diverse protein fold families (Dunwell et al. 2004; Dupont et al. 2010; Valasatava et al. 2018). Alternatively, it could result in a switch to a new metal specificity to achieve the same chemical reaction, of which there are far fewer examples (Emerson et al. 2008; Liu et al. 2024), but is a rational adaptation to altered environmental metal availability (Garcia et al. 2017; Barwinska-Sendra et al. 2020).

We exploited the intensities of the absorption of SodFM cofactors, which report on the oxidation state of the bound metal ions, to assess whether the redox properties of SodFMs followed their trend in catalytic metal preference. In all cases, sampling isozymes across the CR range, enzymatically active metal-loaded forms exhibited nOS values consistent with the sample population containing a mixture of oxidized and reduced metal cofactors at aerobic equilibrium. Inactive forms exhibited nOS values close to zero, implying they contained exclusively reduced metal cofactors. These trends between the nOS of a SodFM and its catalytic metal preference were robust, assembled from biochemical and biophysical measurements obtained from more than 100 separate preparations of purified recombinant protein, and are consistent with prior observations in the literature (Ramilo et al. 1999; Hiraoka et al. 2000; Grove et al. 2008). The inverse relationship between nOS values suggests that evolutionary changes in metal preference of SodFMs follow a predictable biochemical path. For a Mn-preferring isozyme to gain increased catalysis with Fe, it must increase nOS_Fe_. However, because these parameters are intimately linked, increasing nO_Fe_ will necessarily result in decreased nOS_Mn_, thereby reducing its activity with Mn. These properties can be balanced within cambialistic enzymes, in which nOS_Mn_ ≈ nOS_Fe_ and CR ≈ 1. This process occurred in the evolution of *Sa*SodM during its divergence from a Mn-preferring ancestor (Barwinska-Sendra et al. 2020), likely under selection pressure of decreased Mn availability within the mammalian host (Corbin et al. 2008; Garcia et al. 2017). The converse evolutionary process, increasing nOS_Mn_ at the expense of nOS_Fe_, yielded the cambialistic *Bf*SodFM2 from its highly Fe-preferring ancestor (Sendra et al. 2023). Larger changes in nOS can switch metal preference more dramatically, resulting in larger changes in CR, such as those that occurred in the Fe-preferring SodFM1 from candidatus *Wolfebacteria* or the Mn-preferring SodFM2 from *A. tumefaciens* (Sendra et al. 2023). Taken together, the data presented here suggest that SodFM metal preference naturally evolves through a mechanism that is inextricably linked to changes in the metal's oxidation state at rest, through which their catalysis with Mn and Fe are linked.

The predictable biochemical path for evolutionary metal-preference switching might lead to the conclusion that an evolutionary change from one metal specificity to another, for example, from highly Mn-preferring to highly Fe-preferring, must necessarily pass through an intermediate cambialistic evolutionary stage. Indeed, one might even conclude that cambialism, which results from suboptimal tuning and therefore leads to reduced catalytic efficiency with both cofactors, might only ever represent an evolutionary intermediate rather than a positively selected property. The SodFMs are a useful model system with which to interrogate this important evolutionary question, and our data do not support these conclusions. Relatively few mutations are necessary to dramatically alter a SodFM's metal preference (Barwinska-Sendra et al. 2020; Sendra et al. 2023). Just a single mutation in *S. aureus* SodM, converting X_D-2_ to either Thr, Val, or Ile, is sufficient to drive metal preference to become more strongly Fe-preferring (Sendra et al. 2023). Yet, the SodM L_D-2_ residue is conserved in all staphylococcal genomes that possess the *sodM* gene, suggesting cambialism has been selected in *S. aureus*. Although it is not necessary for a full evolutionary switch in preference, i.e. from a Mn-preferring SodFM to become Fe-preferring or vice versa, to transition via a cambialistic evolutionary intermediate, this is likely in at least some cases. Future studies should seek to verify whether cambialism itself has been selected, as suggested in *S. aureus* (Sendra et al. 2023), rather than being a transitional evolutionary state.

Although there are many redox metalloenzymes in which redox tuning has been proposed and explored (Merkens et al. 2008; McIntosh et al. 2014; Beaumet et al. 2022; Eom et al. 2023), the molecular mechanisms by which tuning is achieved by the protein architecture remains unclear. Thus, we lack an understanding of how tuning can be reshaped by evolution to change enzymatic metal preference, or of how it might be synthetically manipulated to create industrially useful metalloenzyme catalysts (Hosseinzadeh et al. 2016; Beaumet et al. 2022; Van Stappen et al. 2022; Jeong et al. 2023). When we introduced mutations into the cofactor's secondary coordination sphere that modulated SodFM metal preference, we observed concomitant changes in spectra and nOS consistent with the hypothesis that the induced metal-preference changes were dependent on modulation of the metal cofactor's resting redox state. These data demonstrate unequivocally that both the nOS and the metal preference of a SodFM are manipulated through changes to the cofactor's secondary coordination sphere. Notably, these mutations involved relatively minor chemical changes in the active site. For example, the change in sidechain properties when swapping of Gly for Leu at position X_D-2_ in the *S. aureus* SodFM pair was sufficient to largely invert their metal preference and their nOS. This is analogous to the minor changes in the hydrophobic sidechains located in the vicinity of the catalytic redox metal ion in the cupin metalloenzyme QueD that have been shown to alter its catalytic metal preference through a redox tuning mechanism (Eom et al. 2023). On the other hand, these hydrophobic changes contrast with the mutations in hydrophilic and charged residues that dramatically alter the reduction potential of electron transfer proteins such as azurin (Hosseinzadeh et al. 2016). The mechanisms by which these minor changes in hydrophobicity manipulate the metal's redox properties are unclear in both QueD and the SodFMs, but may involve altering the local electric field within the active site cavity or altering the spatial positioning of the metal's reactive *d*-orbitals relative to the trajectory of the incoming substrate. Detailed biophysical and structural studies are underway to test such hypothesized mechanisms. Conversely, the SodFM2s more frequently possess Thr, Val, or Ile in the X_D-2_ position (Sendra et al. 2023). Although its mutation to a residue more commonly found in SodFM1s, such as Gly, reduces their CR, it does not fully convert them to Mn-preferring or even fully cambialistic enzymes. Extensive mutagenesis and biophysical studies, analogous to those performed in SodFM1s (Barwinska-Sendra et al. 2020), are needed to determine whether this is due to differences between the active site architecture of these two dominant subfamilies and to characterize the influence of the secondary sphere on catalytic metal preference within the more divergent SodFM3 and SodFM4 subfamilies.

The redox tuning model was proposed to explain the differing cofactor specificity between the pair of isozymes from *E. coli* and has some empirical support in that system (Vance and Miller 1998b; Vance and Miller 2001; Miller 2008). Notably, however, *Ec*SodA was the only isozyme tested herein that was not metalated with Fe when expressed homologously inside aerobic *E. coli* under our standard expression conditions in minimal medium, indicating it displays unusually strong Mn-selective metal-binding properties within this system. Interestingly, selectivity toward the other metal cofactor, Fe, was observed in another SodFM1, *Ba*SodA2, which was unable to bind Mn in vitro or in vivo. These two isozymes are the only SodFMs yet studied that display such metal-binding properties, a phenomenon which warrants further mechanistic investigation. These observations raise the question why such metal selectivity is not more widespread, at least among highly metal-preferring SodFMs where mis-metalation leads to inactive isoforms. Both enzymes can serve as models for future studies of the biochemical mechanisms and physiological selection pressures enabling and driving the novel emergence of metal selectivity in SodFMs. It remains to be studied whether the changes in metal preference in *Bf*SodFM2, *Ec*SodA, or *Ba*SodA2 are specific evolutionary adaptations and, if so, under what selection pressure they emerged. Future studies should determine whether the changes in redox properties observed here, as well as apparent changes in metal selectivity, represent conserved mechanisms by which evolution of catalytic metal usage occur in other redox metalloenzymes.

## Materials and methods

### Bacterial strains and culture conditions

The Fe/Mn SOD-deficient *E. coli* strain, BL21 (λDE3) Δ*sodA*Δ*sodB*, was used as expression host to eliminate *Ec*SodA/*Ec*SodB contamination from recombinant protein preparations (Sendra et al. 2023). *Escherichia coli* strain DH5α was used for molecular biology. BL21 pLysS Δ*sodA*Δ*sodB* was used to improve expression of the wild-type and mutant variants of *Ba*SodA2, which expressed poorly in BL21 Δ*sodA*Δ*sodB*. For selection, 100 µg mL^−1^ ampicillin and 50 µg mL^−1^ kanamycin were added to the growth media.

### Molecular biology

Cloning of the pET22b constructs for expression of *Ba*SodA1, *Ba*SodA2, *Lm*SodA, *Sp*SodA, *Ng*SodB, *Ec*SodA (from *E. coli* B), *Ec*SodB, c*W*Sod, *Am*Sod, and *Bf*Sod was previously described (Sendra et al. 2023). Constructs for the heterologous expression of the genes encoding *Bs*SodA and the wild-type and mutant variants of *Sa*SodA and *Sa*SodM in pET29a produced in a prior study (Barwinska-Sendra et al. 2020) were subcloned into pET22b(+), except for those used for expression of the *Sa*SodA T_D-2_ and *Sa*SodM T_D-2_ mutated constructs, which remained in pET29a. The genes for subcloning were excised from their vector through *Nde*I/*Sac*I (NEB) restriction, purified after electrophoresis (1% w/v agarose) using a gel extraction kit (Sigma-Aldrich), and ligated (T4 Ligase, NEB) into *Nde*I/*Sac*I-digested, Antarctic phosphatase-treated (NEB) pET22b(+) vector. *Escherichia coli* DH5α transformants were ampicillin-selected and screened by PCR using T7 primers. Sequences of the inserts of all constructs were confirmed through DNA sequencing (Eurofins).

### Mutagenesis

Primers for site-directed mutagenesis were designed using the NEBaseChanger tool (NEB) and synthesized (Sigma and IDT). Mutagenesis was performed by PCR using the appropriate primer pairs, treated with KLD enzyme mix (NEB), and then used to transform competent *E. coli* DH5α cells. Transformants were screened by colony PCR. Sequences were confirmed through DNA sequencing.

### Expression and purification of recombinant SODs

Plasmid constructs were transformed into chemically competent *E. coli* BL21(λDE3) Δ*sodA*Δ*sodB* cells and selected on LB agar plates containing ampicillin (pET22) or kanamycin (pET29). A single transformant was inoculated into 50 mL selective media (M9 or LB) and cultured overnight at 37 °C with 180 rpm orbital shaking. The pre-culture was then used to inoculate 0.5 to 1.0 L of fresh selective M9 media supplemented with 1% (w/v) glucose to an OD_600nm_ ∼0.05. For production of Fe-enriched samples of *Ec*SodA only, the cells were cultured in LB medium supplemented with 100 μM Fe. Protein expression was induced by addition of 1 mM isopropyl-β-D-1-thiogalactopyranoside (IPTG), accompanied by addition of either 300 μM ammonium iron sulfate or 1 mM MnCl_2_, followed by incubation at 37 °C for 4 h with 180 rpm orbital shaking. Cells were harvested by centrifugation (4,200 *g*, 25 min, 4 °C), washed in 20 mM Tris pH 7.5, and frozen at −20 °C.

Cell were lysed by sonication on ice in 20 mM Tris pH 7.5, 1× complete EDTA-free protease inhibitor (Roche), 100 µg mL^−1^ lysozyme, 10 µg mL^−1^ DNase, followed by centrifugation at 19,000 g, 4 °C. Cleared cell lysate was subjected to a two-step chromatographic separation using an AKTA (GE Healthcare). Recombinant SODs were initially purified using anion exchange chromatography (Hi Trap Q HP column, GE Healthcare) in 20 mM Tris pH 7.5 buffer with 0 to 1 M NaCl gradient elution. Eluent fractions containing SODs were identified by SDS-PAGE and were pooled and concentrated to a final volume of 1 mL using centrifugal filtration devices (Amicon, 10 kDa cutoff) for subsequent size exclusion chromatography (SEC). For *Ec*SodA, an additional cation exchange step (Hi Trap SP FF column, GE Healthcare) was performed in 20 mM MES pH 5.5 buffer with 0 to 1 M NaCl gradient. *Ec*SodA eluted in the flow-through of both ion exchange steps and was dialyzed overnight at 4 °C in 20 mM Tris pH 7.5, 150 mM NaCl before concentrating for size exclusion chromatography (SEC). SEC was performed in 20 mM Tris pH 7.5, 150 mM NaCl buffer, on a Superdex 200 16/600 GL column (GE Healthcare). SDS-PAGE confirmed that SODs eluted in a single peak at 79 to 86 mL. Protein concentration of recombinant protein preparations was determined by absorbance at 280 nm using each enzyme's theoretical extinction coefficient (ε, ProtParam), and metal loading was analyzed by inductively coupled plasma mass spectrometry (ICP-MS) or inductively coupled plasma optical emission spectrometry (ICP-OES). Protein preparations were split into 0.2 to 1.0 mL aliquots and stored at −20 °C.

Where necessary, for poorly Mn-loaded protein preparations, metal exchange was achieved by unfolding SOD proteins and refolding through dialysis in the presence of 10 mM MnCl_2_ as previously described (Barwinska-Sendra et al. 2020). After dialysis, protein was concentrated using centrifugal filtration devices to 0.5 mL and purified by SEC (Superdex 200 Increase 10/300 column, GE Healthcare) to confirm oligomeric state and remove any aggregated protein and unbound metal ions.

### SOD activity assays

SOD activity was assessed qualitatively using a gel-based negative staining assay (Garcia et al. 2017), or quantitatively using an adaptation of this method in a 96-well plate format, as previously described (Sendra et al. 2023).

### UV/visible spectroscopy

Absorption spectroscopy was performed on a Perkin-Elmer λ35 spectrophotometer, collecting data at room temperature between 200 and 700 nm using quartz cuvettes with a path length of 10 mm (ALS). Measurements of resting spectra (representing the oxidation status of the purified protein when equilibrated under aerobic conditions) were performed in 20 mM Tris pH 7.5, 150 mM NaCl, with samples prepared at 100 µM protein. Each resting sample was split into two, and each half was either chemically oxidized or reduced by incubation with 1 molar equivalent of potassium permanganate or 3 molar equivalents of sodium dithionite for 10 min, respectively, followed by extensive buffer exchange using centrifugal filtration in 20 mM Tris pH 7.5, 150 mM NaCl. Spectra were converted to extinction coefficient, ε, using protein concentration and were normalized to nOS for comparison between isozymes as follows:


$$\frac{\mathrm{resting}\;\mathrm{peak}\;\mathrm{intensity}-\mathrm{reduced}\;\mathrm{peak}\;\mathrm{intensity}}{\mathrm{oxidised}\;\mathrm{peak}\;\mathrm{intensity}-\mathrm{reduced}\;\mathrm{peak}\;\mathrm{intensity}}$$

where peak intensity corresponds to absorbance at 480 nm or 350 nm for Mn-loaded and Fe-loaded enzymes, respectively. In order to test whether the resting state could be enzymatically regenerated after complete oxidation or reduction, the buffer-exchanged oxidized and reduced forms of SOD were mixed in an equimolar ratio and incubated with 0.1 mM riboflavin for 20 min on a white light box, before extensive buffer exchange to remove the riboflavin. UV-visible spectra showed that the resting state was regenerated, demonstrating enzymatic activity had not been lost through chemical oxidation or reduction.

### Elemental analysis

Aliquots of purified protein samples (20 μM) in 20 mM Tris pH 7.5, 150 mM NaCl, were each diluted to 5 mL with 2% HNO_3_ for elemental analysis. Elemental composition of the resulting acid solutions was quantified using an iCAP RQ ICP-MS (Thermo Fisher Scientific) instrument (University of Plymouth Enterprise Ltd) or using an iCAP Pro ICP-OES (Thermo Fisher Scientific) instrument, as previously described (Sendra et al. 2023).

### X-ray crystallography

Protein preparations for crystallography were concentrated to 15 to 19 mg mL^−1^ and were subjected to crystallization screening using a Mosquito liquid handling robot (TTP Labtech) with commercially available matrix screens: PACT, JCSG+, Structure, Morpheus (Molecular Dimensions), and Index (Hampton Research) in 96-well MRC crystallization plates (Molecular Dimensions) using the sitting drop of vapor-diffusion method (two drops per well, containing 100 nL + 100 and 200 nL + 100 nL of protein and crystallization liquor, respectively), incubated at 20 °C. *Ng*SodB crystallized in Morpheus condition D1 (0.1 M MES/imidazole pH 6.5, 10% w/v PEG 20000, 20% v/v PEG MME 550, 0.02 M of each additive alcohol: 1,6-hexanediol, 1-butanol, (RS)-1,2-propanediol, 2-propanol, 1,4-butanediol, 1,3-propanediol), *Lm*SodA crystallized in PACT condition A8 (0.2 M Ammonium chloride, 0.1 M Sodium acetate pH 5.0, 20% w/v PEG 6000), and *Lm*SodA V_D-2_-I_D-1_ crystallized in structure condition D6 (0.2 M ammonium sulfate, 30% w/v PEG 8000). Crystals were harvested in 20% PEG-400 cryoprotectant and flash-frozen in liquid nitrogen. X-ray diffraction data were collected at the Diamond Light Source synchrotron (Didcot, UK) on beamline I03 and beamline I24 at 100 K on two trips: mx24948-132 and mx24948-124. Structural solution and model building were as described previously (Sendra et al. 2023). 3LIO was used as a search model for molecular replacement for *Ng*SodB, and 2RCV was used as a search model for *Lm*SodA. The generated structural model of *Lm*SodA was subsequently used to solve the structure of the *Lm*SodA mutant variant.

### Evolutionary analysis of *Staphylococcus* SodFM1 homologs

All 21,452 available NCBI RefSeq *Staphylococcaceae* genome assemblies (excluding atypical) were downloaded on 2024 December 1. SodFM homologs were identified using PFAM (Mistry et al. 2021) hidden Markov model (HMM) profiles (Sod_Fe_C, PF02777.21; Sod_Fe_N, PF00081.2) with hmmsearch (Eddy 2011) (-E 1e^−5^) profile search implemented in HMMER3.3. HMM search identified 37,603 *Staphylococcus* SodFM homologs including 478 unique protein sequences encoded by 1051 unique nucleotide sequences. Of the 1051 nucleotide sequences, 73 shared less than 98% pairwise sequence identity (mean of 86% for protein and 83% for nucleotide sequences) with each other and only these 73 sequences were used in the subsequent analyses. The protein sequences were aligned with MAFFT (Katoh and Standley 2013), and corresponding nucleotide alignments were generated with local version (Suyama et al. 2006) of PAL2NAL_v14.

Pairwise sequence identity was calculated with trimAl (Capella-Gutiérrez et al. 2009) (-sident). Phylogenies were reconstructed using IQ-TREE (Nguyen et al. 2015) with 1,000 ultrafast bootstraps (Hoang et al. 2018). Nucleotide *Staphylococcaceae* SodFM tree was generated under best fitting GTR + F + R4 model, and protein *Staphylococcaceae* SodFM tree was generated under best fitting LG + G4 model selected with ModelFinder (Kalyaanamoorthy et al. 2017) according to Bayesian information criterion implemented in IQ-TREE. Bootstrap values and branch lengths were removed in R using ape library (Paradis and Schliep 2019), and the phylogenies and the corresponding nucleotide alignments were used in the subsequent analyses. Positively and negatively selected sites were inferred with FUBAR (Murrell et al. 2013). Gene-wide positive selection at least one site on at least one branch was tested with BUSTED (Wisotsky et al. 2020). Specific sites under episodic positive selection were inferred with MEME (Murrell et al. 2015).

RELAX (Wertheim et al. 2015) analysis was used to test trends in the relaxation or intensification of the strength of natural selection along specified set of test branches relative to the specified background branches.

### Bioinformatic identification of *Bacillus* SodFM homologs

All 11,394 available NCBI RefSeq Bacillaceae genome assemblies (excluding atypical) were downloaded on 2023 December 12. HMM searches with hmmsearch (-E 1e^−5^) identified 23,278 SodFM homologs in *Bacillaceae* including 8,958 SodFM3 and 14,320 SodFM1 redundant sequences (i.e. including identical sequences encoded across, e.g. multiple *B. anthracis* genome assemblies). The identified SodFMs included 2,740 unique protein sequences of which 1,137 were SodFM3s and 1,603 SodFM1s. SodFM1 subfamily was subdivided into 1,250 SodA1 (9,868 redundant) and 353 SodA2 (4,452 redundant) unique protein sequences based on their protein tree topology and Xd-2 residue identity verified in MAFFT alignments.

*Bacillaceae* species tree was generated using concatenated alignment of seven universally conserved orthologues as described previously (Sendra et al. 2023). To reduce the oversampling of highly identical genome assemblies from some lineages (e.g. multiple *B. cereus/anthracis* isolates), only those containing unique sequences of all seven universally conserved orthologues were used, resulting in the final dataset of 1,115 genomes encoding 111 SodA2s, 1,185 SodA1s, and 819 SodFM3s.

Firmicutes dataset included 828 representative genome assemblies sampled from across all major Firmicutes lineages. HMM searches with hmmsearch (-E 1e^−5^) identified 2,153 (2,759 total) unique SodFMs including 1,359 unique (1,772 total) SodFM1s in Firmicutes genomes. *Bacillaceae* and Firmicutes SodFM protein trees were generated using IQ-TREE with 1,000 ultrafast bootstraps under WAG + G4 model.

### Other bioinformatics methods

Multiple sequence alignments were inspected using Jalview (Waterhouse et al. 2009). Phylogenetic trees were inspected in Archaeopteryx (Han and Zmasek 2009). The phylogenies were visualized and annotated in R with ggplot2 (Villanueva and Chen 2019), ggtree (Yu et al. 2017), ape (Paradis and Schliep 2019), treeio (Wang et al. 2020), ggtreeExtra (Xu et al. 2021), and ggnewscale.

## Supplementary Material

msag040_Supplementary_Data

## Data Availability

Source data underpinning this study is included in the Source Data file, available online. All additional raw data will be available on request. Computational analyses used publicly available data derived from NCBI. The crystallography data is available from the Protein Databank (PDB).
