# Supplementary material for: An enzyme's metal preference evolves through redox modulation driven by the cofactor's secondary coordination sphere
Source: Mol Biol Evol. 2026 Feb 13;43(3):msag040. doi: 10.1093/molbev/msag040 (PMC13008324; doi:10.1093/molbev/msag040)
Supplement: msag040_Supplementary_Data [file msag040_supplementary_data.zip › Supplementary-data-revised.pdf]

SUPPLEMENTARY INFORMATION

An enzyme's metal preference evolves through redox modulation driven by the cofactor's secondary coordination sphere

ES Mackenzie[1], KM Sendra[1], A Baslé[1], R Mazgaj[2], TE Kehl-Fie[3,4,5]*, KJ Waldron[1,2]*.

[1]Biosciences Institute, Faculty of Medical Sciences, Newcastle University, Newcastle upon Tyne, NE2 4HH, United Kingdom.

[2]Institute of Biochemistry and Biophysics, Polish Academy of Sciences, Pawińskiego 5a, 02-106 Warsaw, Poland.

[3]Department of Microbiology, University of Illinois Urbana-Champaign, Urbana, IL, 61801, USA.

[4]Carl R. Woese Institute for Genomic Biology, University of Illinois Urbana-Champaign, Urbana, IL, 61801, USA.

[5]Department of Microbiology & Immunology, University of Iowa, Iowa City, IA, 52242, USA.

*Authors to whom correspondence should be addressed:

Thomas E. Kehl-Fie, email: thomas-kehl-fie@uiowa.edu.

Kevin J. Waldron, email: kwaldron@ibb.waw.pl.

Classification: Biological Sciences/Biochemistry

Keywords: Metalloenzyme, Protein evolution

```
cWSod (SodFM1) --------------MKHELPPLPYAYNALEPFIDAKTLEIHHTKHHQSYVDKLNAALEKY 46
EcSodA    (FM1) --------------MSYTLPSLPYAYDALEPHFDKQTMEIHHTKHHQTYVNNANAALESL 46
SaSodM    (FM1) --------------MAFKLPNLPYAYDALEPYIDQRTMEFHHDKHHNTYVTKLNATVEGT 46
SaSodA    (FM1) --------------MAFELPKLPYAFDALEPHFDKETMEIHHDRHHNTYVTKLNAAVEGT 46
SpSodA    (FM1) --------------MAFELPKLPYSYDALEPHIDKETMEIHHSKHHNTYVTKLNAAVEGT 46
LmSodA    (FM1) --------------MTYELPKLPYTYDALEPNFDKETMEIHYTKHHNTYVTKLNEAVAGH 46
BaSodA1   (FM1) -------------MAKHELPNLPYAYDALEPHFDKETMNIHHTKHHNTYITNLNAALEGH 47
BaSodA2   (FM1) -------------MSSFQLPKLSYDYDELEPYIDSNTLSIHHGKHHATYVNNLNAALENY 47
BsSodA    (FM1) --------------MAYELPELPYAYDALEPHIDKETMTIHHTKHHNTYVTNLNKAVEGN 46
BfSod (SodFM2) ---------------MYEMPKLPYANNALEPVISQQTIDYHYGKHLQTYVNNLNSLVPGT 45
EcSodB    (FM2) --------------MSFELPALPYAKDALAPHISAETIEYHYGKHHQTYVTNLNNLIKGT 46
NgSodB    (FM2) --------------MEHKLPQLPYGPDALSPHLSKETLEFHYGKHHQTYITNLNNQIKGT 46
AmSod (SodFM3) MTKQQAPSTIGYADGKYVLPPLPYAYDALEPMLDEKTVRIHHDKHHAAYVAGANAAAEKL 60
                              . :* * *  : * * :. .*: *: :*  :*:   *

cWSod (SodFM1) PDLQDKTVEELIKSLNELPEEIRTTVRNNAGGHFSHTLYWNIMNPAT-QEYIPEELGNAL 105
EcSodA    (FM1) PEFANLPVEELITKLDQLPADKKTVLRNNAGGHANHSLFWKGLKKGT-T--LQGDLKAAI 103
SaSodM    (FM1) -ELEHQSLADMIANLDKVPEAMRMSVRNNGGGHFNHSLFWEILSPNS-E--EKGGVIDDI 102
SaSodA    (FM1) -DLESKSIEEIVANLDSVPANIQTAVRNNGGGHLNHSLFWELLSPNS-E--EKGTVVEKI 102
SpSodA    (FM1) -EFENKSLEDLIANLDSVPENLRTAVRNNGGGHLNHSIYWQILAPNS-E--EKGEVVDKI 102
LmSodA    (FM1) PELASKSAEELVTNLDSVPEDIRGAVRNHGGGHANHTLFWSILSPNG-GGAPTGNLKAAI 105
BaSodA1   (FM1) AELADKSVEELVANLNEVPEAIRTAVRNNGGGHANHTFFWTILSPNG-GGQPVGELATAI 106
BaSodA2   (FM1) SELHNKSLEELLCNLETLPKEIVTAVRNNGGGHYCHSLFWEVMSPRG-GGEPNGDVAKVI 106
BsSodA    (FM1) TALANKSVEELVADLDSVPENIRTAVRNNGGGHANHKLFWTLLSPNG-GGEPTGALAEEI 105
BfSod (SodFM2) -EYEGKTVEAIVASA------PDGAIFNNAGQVLNHTLYFLQFAPKPAKNEPAGKLGEAI 98
EcSodB    (FM2) -AFEGKSLEEIIRSS-------EGGVFNNAAQVWNHTFYWNCLAPNA-GGEPTGKVAEAI 97
NgSodB    (FM2) -EFENLPLEEIVKKS-------SGGVFNNAAQTWNHTFYWLGFTPKG-QGKPAGELAAAI 97
AmSod (SodFM3) REIADGKLDASAT------TNWVRNLSFNASGHVLHTIYWTNMTPDP-KKEPQGPLVDAI 113
                                       :  :..     *.:::  :             :   :

cWSod (SodFM1) VETFGSIIAFKEQFSKAAANIFGSGWVWLAADANKKLKIVSTTGHDNPLMT------GDA 159
EcSodA    (FM1) ERDFGSVDNFKAEFEKAAASRFGSGWAWLVLK-GDKLAVVSTANQDSPLMGEAISGASGF 162
SaSodM    (FM1) KAQWGTLDEFKNEFANKATTLFGSGWTWLVVN-DGKLEIVTTPNQDNPLTE------GKT 155
SaSodA    (FM1) KEQWGSLEEFKKEFADKAAARFGSGWAWLVVN-NGQLEIVTTPNQDNPLTE------GKT 155
SpSodA    (FM1) KEQWGSLDEFKSEFADKAAGRFGSGWAWLVVN-NGKLEIVTTANQDSPLTD------GLT 155
LmSodA    (FM1) ESEFGTFDEFKEKFNAAAAARFGSGWAWLVVN-DGKLEIVSTANQDSPLSD------GKT 158
BaSodA1   (FM1) EAKFGSFDAFKEEFAKAGATRFGSGWAWLVVN-NGELEVTSTPNQDSPLTE------GKT 159
BaSodA2   (FM1) DYYFNTFDNLKDQLSKAAISRFGSGYGWLVLD-GEELSVMSTPNQDTPLQE------GKI 159
BsSodA    (FM1) NSVFGSFDKFKEQFAAAAAGRFGSGWAWLVVN-NGKLEITSTPNQDSPLSE------GKT 158
BfSod (SodFM2) KRDFGSFENFKKEFNAASVGLFGSGWAWLSVDKDGKLHITKEPNGSNPVRA------GLK 152
EcSodB    (FM2) AASFGSFADFKAQFTDAAIKNFGSGWTWLVKNSDGKLAIVSTSNAGTPLTT------DAT 151
NgSodB    (FM2) DAKWGSFEKFQEAFNACAAGTFGSGWAWLVKTPVGGLDLISTSNAATPLTT------ENT 151
AmSod (SodFM3) KEKFGSLEAMMKEFKAASQGVEGSGWGILGVDPISKTLVICGAEKHQNLE-----IPGLV 168
                          :.:.  :    :    .     ***:  *          :          :

cWSod (SodFM1) PLMVIDIWEHAYYLHYQNRRPEYIENWWNVLDWKAVEERYNALG-----     203
EcSodA    (FM1) PIMGLDVWEHAYYLKFQNRRPDYIKEFWNVVNWDEAAARFAAKK-----     206
SaSodM    (FM1) PILLFDVWEHAYYLKYQNKRPDYMTAFWNIVNWKKVDELYQAAK-----     199
SaSodA    (FM1) PILGLDVWEHAYYLKYQNKRPDYIGAFWNVVNWEKVDELYNATK-----     199
SpSodA    (FM1) PILALDVWEHAYYLKFQNKRPDYISTFWNVVNWEKVDELYKAAK-----     199
LmSodA    (FM1) PVLGLDVWEHAYYLKFQNRRPEYIETFWNVINWDEANKRFDAAK-----     202
BaSodA1   (FM1) PVIGLDVWEHAYYLNYQNRRPDYIGAFWNVVDWNAAEKRYQEAK-----     203
BaSodA2   (FM1) PLLVIDVWEHAYYLKYQNRRPEFVTNWWHTVNWDRVNEKYLQAIQSQKH     208
BsSodA    (FM1) PILGLDVWEHAYYLNYQNRRPDYISAFWNVVNWDEVARLYSEAK-----     202
BfSod (SodFM2) PLLGFDVWEHAYYLDYQNRRADHVNKLWEIIDWDVVEKRL---------     192
EcSodB    (FM2) PLLTVDVWEHAYYIDYRNARPGYLEHFWALVNWEFVAKNLAA-------     193
NgSodB    (FM2) PLLTCDVWEHAYYIDYRNSRPNYLKGFWEIVNWDEVAKRFAA-------     193
AmSod (SodFM3) PILVCDVWEHAYYLKHQNLRADYIEDFCRLINWDDVERRYQEAMAS---     214
                *::   *:******:..:* *   .:      ::*. .
```

Supplementary Figure S1. Sequence alignment of analysed SodFM isozymes.

Sequence alignment, produced using Clustal Omega, of the protein sequences of all SodFM isozymes tested within this study. Metal ligands are highlighted orange, and the X_{D-2} and X_{D-2} residues are highlighted green.

*Am*Sod	(SodFM3)	100.0	40.3	36.4	42.1	39.1	39.6	43.8	39.5	42.4	42.6	41.0	32.6	35.3
c*W*Sod	(SodFM1)	40.3	100.0	48.0	51.0	46.2	49.3	50.3	50.5	55.5	51.5	41.9	43.5	39.4
*Ba*SodA2	(SodFM1)	36.4	48.0	100.0	49.5	50.3	50.3	49.3	51.0	53.7	55.5	38.4	42.7	38.5
*Ec*SodA	(SodFM1)	42.1	51.0	49.5	100.0	49.3	54.8	57.3	62.5	59.5	60.5	42.0	45.3	47.4
*Sa*SodM	(SodFM1)	39.1	46.2	50.3	49.3	100.0	74.9	75.4	59.3	60.8	62.8	43.1	49.0	43.2
*Sa*SodA	(SodFM1)	39.6	49.3	50.3	54.8	74.9	100.0	81.9	65.3	66.3	71.9	45.7	50.5	45.3
*Sp*SodA	(SodFM1)	43.8	50.3	49.3	57.3	75.4	81.9	100.0	66.8	64.3	70.4	44.2	52.1	46.8
*Lm*SodA	(SodFM1)	39.5	50.5	51.0	62.5	59.3	65.3	66.8	100.0	70.8	73.8	46.8	50.5	49.5
*Ba*SodA1	(SodFM1)	42.4	55.5	53.7	59.5	60.8	66.3	64.3	70.8	100.0	76.7	45.8	48.4	49.5
*Bs*SodA	(SodFM1)	42.6	51.5	55.5	60.5	62.8	71.9	70.4	73.8	76.7	100.0	49.0	53.1	49.5
*Bf*Sod	(SodFM2)	41.0	41.9	38.4	42.0	43.1	45.7	44.2	46.8	45.8	49.0	100.0	51.1	47.9
*Ec*SodB	(SodFM2)	32.6	43.5	42.7	45.3	49.0	50.5	52.1	50.5	48.4	53.1	51.1	100.0	63.7
*Ng*SodB	(SodFM2)	35.3	39.4	38.5	47.4	43.2	45.3	46.8	49.5	49.5	49.4	47.9	63.7	100.0

Supplementary Figure S2. Sequence identity matrix of analysed SodFM isozymes.

Sequence identity matrix, produced using Clustal Omega, of the protein sequences of all SodFM isozymes tested within this study.

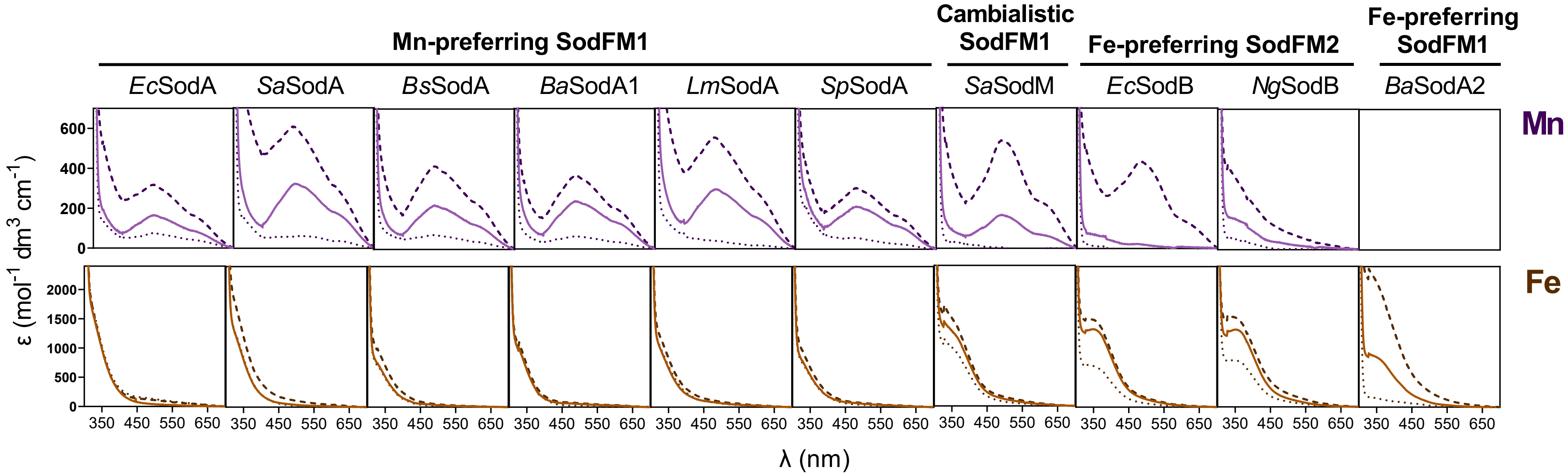

Supplementary Figure S3. Spectra of wild type SodFMs at rest.

UV-visible absorption spectra of all wild type SodFMs analysed in triplicate in this study when Mn-loaded (upper panels, purple lines) and Fe-loaded (lower panels, brown lines). Resting spectra of proteins equilibrated at atmospheric conditions are shown as a solid line, spectra of oxidised and reduced proteins are shown as darker dashed and dotted lines, respectively. This analysis demonstrated that the resting spectra lie between oxidised and reduced spectra for catalytically active forms (i.e. when Mn-bound for Mn-preferring SodFM1s, when Fe-bound for Fe-preferring SodFM2s, and in both metal forms for cambialistic *Sa*SodM) but are similar to the reduced spectra for inactive forms. Spectra are shown as $n = 1$, but are representative of further replicates ($n = 3$). As we were unable to generate the Fe-loaded form of *Ec*SodA nor the Mn-loaded form of *Ba*SodA2, no corresponding spectra were obtained for these forms.

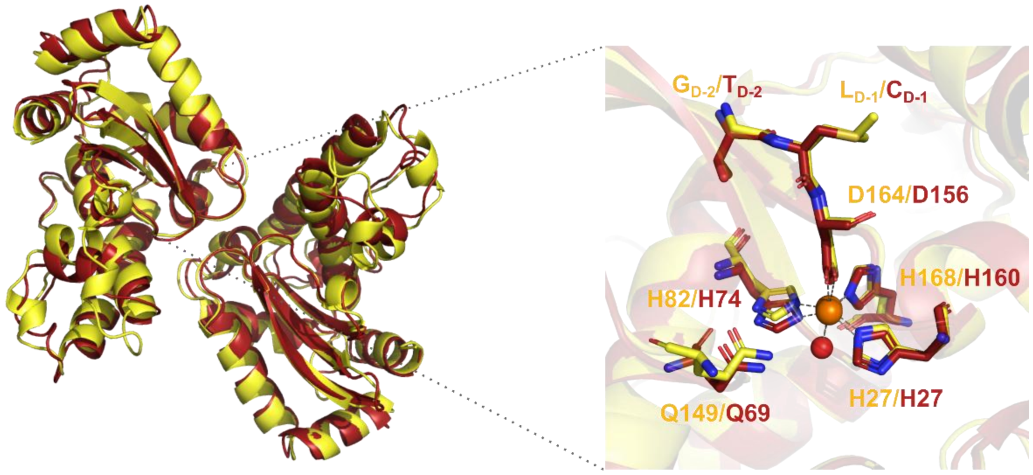

Supplementary Figure S4. Structural comparison of the SodFM1 subfamily isozyme, *Lm*SodA, and the SodFM2 subfamily isozyme, *Ng*SodB.

Overlaid structural models derived from X-ray crystallography showing the homodimeric assemblies (left) and zoomed in view of the active sites (right panel) of the Mn-preferring SodFM1 isozyme from *Listeria monocytogenes* (yellow) and the Fe-preferring SodFM from *Neisseria gonorrhoeae* (red). Within the active sites (right), key amino acid residues are shown in ball-and-stick representation: the metal binding ligands (H27 in both, H82 in *Lm*SodA and H74 in *Ng*SodB, H168 in *Lm*SodA and H160 in *Ng*SodB, and D164 in *Lm*SodA and D156 in *Ng*SodB), the water-coordinating residue (Q149 in *Lm*SodA and Q69 in *Ng*SodB), and the two second sphere residues that were targeted for mutagenesis herein, X_{D-2} (G162 in *Lm*SodA and T154 in *Ng*SodB) and X_{D-1} (L163 in *Lm*SodA and C155 in *Ng*SodB). The metal ions are shown as orange spheres and a solvent molecule is indicated with a red sphere. Despite these isozymes being from distinct subfamilies (47.9% sequence identity), their overall structures are remarkably similar, including the position of an important Gln residue which is encoded in distinct sequence loci in these two subfamilies.

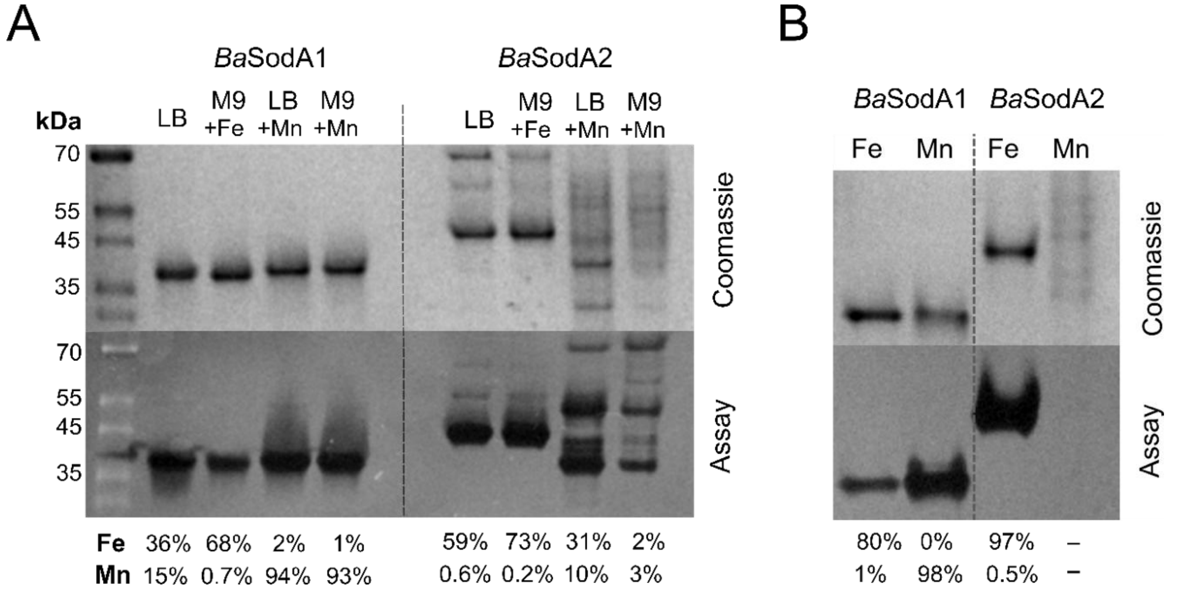

Supplementary Figure S5. No significant Mn-incorporation by *Ba*SodA2 was achieved through metal-supplementation of expression media *or in vitro* unfolding/refolding dialysis against excess Mn.

A Native-PAGE (upper panels) and in-gel SOD activity assays (lower panels) from analyses of purified preparations of *Ba*SodA1 (left) and *Ba*SodA2 (right) after expression either in rich (LB) or minimal (M9) media, without added metals or when supplemented with either 300 µM ammonium iron sulphate or 1 mM manganese chloride. The results from elemental analysis by ICP-MS reporting the percentage of Fe and Mn incorporation into the enzymes are annotated below. While *Ba*SodA1 could be loaded to high levels with either metal through overexpression in the presence of excess metal ion, expression of *Ba*SodA2 in Mn-supplemented media resulted in low yields of protein, which was found to be unstable, with no significant Mn-loading. Attempts to optimise expression conditions, including altering expression strain and incubation time and temperature after induction, also resulted in insignificant Mn-loading. **B** Native-PAGE and in-gel SOD activity assays from analyses of purified preparations of Fe-loaded *Ba*SodA1 and *Ba*SodA2 and of these enzymes after *in vitro* unfolding/refolding dialysis against 10 mM $MnCl_2$. The refolding procedure achieved 98% Mn-loading of *Ba*SodA1, but no soluble *Ba*SodA2 remained for analysis due to total protein precipitation. In the attempt to yield soluble Mn-bound *Ba*SodA2 conditions were varied for optimisation, including protein concentration, $MnCl_2$ concentration, $NaCl_2$ concentration, addition of dithiothreitol to dialysis buffers and incubation time in each dialysis step. None of the conditions tested resulted in enough protein recovery for elemental analysis or spectral analysis.

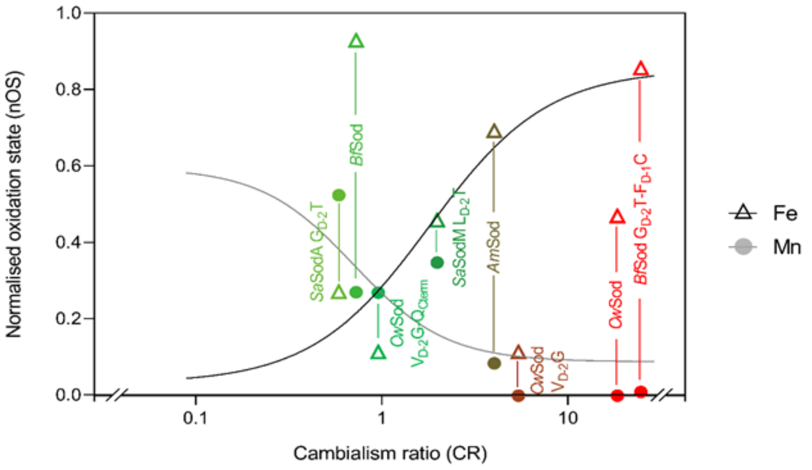

Supplementary Figure S6. Correlation of cambialism and oxidation state extends across more divergent SODs.

Normalised oxidation state (nOS) values of a further set of phylogenetically and functionally divergent SODs when Fe-loaded (open triangles) and Mn-loaded (closed circles) compared with cambialism ratio. Broadly, these SODs follow the same trend as the core dataset, despite being more evolutionarily and functionally divergent. These SODs were measured as n = 1 and were not used in the calculation of the trendline shown, which is calculated from the triplicate data shown in Figure 6. Data points are coloured by cambialism ratio and are annotated with the corresponding enzyme name.

Data statistics			
	***Ng*SodB**	***Lm*SodA**	***Lm*SodA $G_{D-2}V$-$L_{D-1}I$**
Beamline	I24	I03	I03
Date	25/02/2022	22/11/2021	22/11/2021
Wavelength (Å)	1.00	0.898	0.898
Resolution (Å)	67.47 – 2.00 (2.03 – 2.00)	52.72 – 1.65 (1.68 – 1.65)	51.99 – 1.40 (1.42 – 1.40)
Space group	$P2_12_12_1$	$P2_12_12_1$	$P2_12_12_1$
**Unit-cell parameters**			
a, b, c (Å)	104.2, 105.6, 162.6	52.7, 94.1, 94.5	52.0, 93.4, 94.9
α, β, γ (°)	90.0, 90.0, 90.0	90.0, 90.0, 90.0	90.0, 90.0, 90.0
Unit-cell volume ($Å^3$)	447110	117215	115144
Solvent content (%)	46.4	52.6	51.6
Total reflections	1596241 (79285)	650576 (28715)	1211451 (42183)
Unique reflections	121479 (5956)	57150 (2727)	91660 (4487)
$CC_{1/2}$	1.00 (0.44)	0.97 (0.54)	1.00 (0.54)
Completeness (%)	100 (100)	99.7 (99.4)	100 (100)
Multiplicity	13.10 (13.30)	11.40 (10.50)	13.20 (9.40)
Rmerge	0.16 (2.89)	0.16 (3.17)	0.07 (2.06)
I/σ(I)	10.00 (0.90)	7.00 (0.90)	16.70 (1.00)
PDB ID	9TWC	9TW9	9TWA

Table S1: Summary of X-ray crystallography data collection statistics.

Summary of parameters of the datasets collected for *Ng*SodB, *Lm*SodA and *Lm*SodA $G_{D-2}V$-$L_{D-1}I$ protein crystals. Values in parenthesis are for the highest resolution shell.
